# Implant selection at elective aseptic revision of a primary total knee replacement: A scoping review and map of the evidence

**DOI:** 10.1371/journal.pone.0361040

**Published:** 2026-10-21

**Authors:** Ben Tyas, John Norman, Shivam Nitin Kolhe, Melissa Harden, Catherine Hewitt, David Deehan, Paul Baker

**Affiliations:** 1 Department of Health Sciences, University of York, York, United Kingdom; 2 Academic Centre for Surgery, University Hospitals Tees, Middlesbrough, United Kingdom; 3 Trauma & Orthopaedics, Health Education England Yorkshire and the Humber, Leeds, United Kingdom; 4 Centre for Reviews and Dissemination, University of York, York, United Kingdom; 5 Trauma & Orthopaedics, Newcastle Hospitals NHS Foundation Trust, Newcastle Upon Tyne, United Kingdom; 6 Faculty of Medical Sciences, Newcastle University, Newcastle Upon Tyne, United Kingdom; 7 School of Health & Life Sciences, Teesside University, Middlesbrough, United Kingdom; Shuguang Hospital Affiliated to Shanghai University of Traditional Chinese Medicine, CHINA

## Abstract

**Purpose:**

The recent James Lind Alliance (JLA) Priority Setting Partnership (PSP) on ‘the problematic knee replacement’ highlighted implant selection at revision knee arthroplasty (rTKA) as a ‘Top 10’ research priority. The aims of this scoping review are to map the evidence for the implants used at first-time elective aseptic rTKA, to identify gaps in the evidence-base, and to provide recommendations for future research.

**Methods:**

A scoping review was conducted in accordance with the Arksey and O’Malley framework and the Joanna Briggs Institute Manual for Evidence Synthesis, and reported according to the PRISMA extension for Scoping Reviews. A comprehensive search of MEDLINE, Embase, CINAHL, CDSR, CENTRAL, DARE, HTA, SCI, PROSPERO and KSR Evidence was conducted on 17^th^ August 2026, covering studies published between 2000 and 2026. Annual reports of 17 national joint replacement registries (NJRRs) were also sought. The review was registered on the Open Science Framework (https://doi.org/10.17605/OSF.IO/SD49V).

**Results:**

Following deduplication, 7,096 research articles and 155 NJRR reports were identified. After applying eligibility criteria, 121 research articles and six NJRR reports were included. Twenty-two studies (18%) had cohorts clearly defined as exclusively comprising first-time elective aseptic rTKA procedures. The evidence consisted mostly of retrospective case series (45/121, 37%) and retrospective comparative studies (41/121, 34%). Most evidence was Level IV (48/121, 40%) or Level III (46/121, 38%) with no Level I evidence identified. Approximately half the studies were from the United States (58/102, 48%). Five studies (4%) reported mean or median follow up ≥10 years. A map of the evidence, tabulated by five key themes, is presented.

**Conclusions:**

There is a lack of high-level comparative evidence to support implant selection at first-time elective aseptic rTKA. Future research should focus on more clinically homogeneous cohorts and compare outcomes associated with cemented versus hybrid/cementless stems, constraint levels, and metaphyseal fixation devices.

## Introduction

Revision knee arthroplasty (rTKA) is a technically challenging procedure associated with higher complication rates, inferior implant survivorship, and greater resource utilisation compared to primary knee replacement surgery [1–3]. According to data from the National Joint Registry, the annual number of rTKA procedures increased 149% between 2006 and 2019, with the most recent data showing 6,460 rTKAs were performed in 2024 [4,5]. Approximately 70% of all rTKA procedures are elective aseptic revisions of primary knee replacements [4].

In 2020, the James Lind Alliance (JLA) conducted a Priority Setting Partnership (PSP) to explore unanswered research questions about the assessment, management and rehabilitation of ‘problematic knee replacements’ [6]. Implant selection in revision knee surgery was highlighted as one of the ‘Top 10’ research priorities. The British Association for Surgery of the Knee (BASK) has published ‘A guide to good practice for revision knee replacement’, however, recognising the lack of evidence to support choice of implants, the guidance provided in this domain is limited [7].

The zonal fixation framework is widely used to guide implant selection in rTKA [8]. This framework proposes that there are three ‘zones’ in both the femur and tibia in which implant fixation can be achieved: Zone 1, the joint surface or epiphysis; Zone 2, the metaphysis and Zone 3, the diaphysis. There is an increasing use of metaphyseal cones and sleeves to achieve Zone 2 fixation, whilst intramedullary stems may be implanted using a fully cemented or hybrid technique to achieve Zone 3 fixation [9–11]. Additionally, choice of constraint must be considered, where the use of higher degrees of constraint to address ligament insufficiency and bone loss might be weighed against the risks of excessive stress at the fixation interface [12,13].

Several systematic reviews have attempted to provide guidance on implant selection in rTKA [11,14–33]. However, the authors of nearly all of these reviews highlight the challenges of navigating literature in this field, often finding a predominance of heterogeneous low-quality evidence. By drawing on heterogeneous literature, scoping reviews are particularly effective in this setting, as they help to consolidate knowledge across fragmented areas of study [34]. The aims of this scoping review are to map the evidence for the implants used at elective aseptic revision of a primary total knee replacement (pTKA), to identify gaps in the current knowledge base, and to provide recommendations for future research.

## Methods

A scoping review was conducted in accordance with the Arksey and O’Malley framework and the Joanna Briggs Institute (JBI) Manual for Evidence Synthesis, and reported according to the Preferred Reporting Items for Systematic reviews and Meta-Analyses extension for Scoping Reviews (PRISMA-ScR) [34–36]. A protocol for the review was published *a priori* and is publicly available on the Open Science Framework (https://doi.org/10.17605/OSF.IO/SD49V) [37].

The primary research question was developed by consensus amongst authors as follows: ‘*What is the evidence to support the implants used at elective aseptic revision of a primary total knee replacement?’*

The search strategy and final eligibility criteria were developed iteratively through a piloting phase, as detailed in the protocol [37]. In summary, the search comprised of two parts: 1) a search of 10 electronic databases for published literature and 2) a search of annual reports from 17 national joint replacement registries (NJRRs).

A search of MEDLINE, Embase, Cumulative Index to Nursing and Allied Health Literature (CINAHL), Cochrane Database of Systematic Reviews (CDSR), Cochrane Central Register of Controlled Trials (CENTRAL), Database of Abstracts of Reviews and Effects (DARE), Health Technology Assessment (HTA) database, Science Citation Index (SCI), Prospective Register of Systematic Reviews (PROSPERO) and Kleijnen Systematic Reviews Evidence (KSR Evidence) was conducted on 17^th^ August 2026. Searches were limited to studies in English language published between 2000–2026 and case reports, editorials and letters were excluded. The MEDLINE search strategy is provided in Supporting Information File 1; all other search strategies are available in the protocol [37]. Supplementary forward and backward citation checking of relevant included studies was undertaken to identify additional eligible studies. The inclusion and exclusion criteria are shown in Table 1. Non-randomised studies with fewer than 100 cases were excluded; this threshold was selected pragmatically during pilot screening to exclude small case series, which were numerous in this literature, and to focus the review on larger observational studies while maintaining a feasible scope. A 30% threshold was similarly applied to exclude studies in which a substantial proportion of the cohort comprised revisions for infection or periprosthetic fracture, multiply revised knees, or other procedures outside the review scope, while allowing inclusion of mixed cohorts in which elective aseptic revision of a primary TKA remained the predominant population. Where the indications for revision or details of previous surgeries were not clearly reported, studies were retained if the available information suggested that the majority of cases were elective aseptic revisions of pTKAs. Among the included studies, we additionally identified those in which the study cohort was clearly defined as exclusively comprising first-time elective aseptic rTKA procedures.

**Table 1:** Inclusion and exclusion criteria for research articles identified from electronic databases.

Inclusion criteria	Exclusion criteria
• Studies investigating patients undergoing elective aseptic revision of a primary total knee replacement • Interventions or comparators consisting at least one of: constraint, metal augments, metaphyseal fixation devices, stem design, fixation methods or bearing design. • Outcomes including at least one of: implant survivorship, re-revision, patient-reported outcomes measures, patient-reported symptoms	• Level V evidence including narrative literature reviews • Non-randomised studies with <100 cases • In-vitro/non-human/retrieval studies • Studies with >30% revisions for acute/chronic infection or periprosthetic fracture • Studies with >30% multiply revised knees • Studies with >30% cases of revision of unicompartmental knee replacements/secondary patella resurfacing/arthrodesis/amputation

Citations were imported into Covidence systematic review software (Veritas Health Innovation, Melbourne, Australia; web-based platform, accessed 2025–2026) for deduplication and screening. All initial titles/abstracts and subsequent full texts were screened by two reviewers independently. Conflicts were resolved by consensus or, where necessary, by final decision from one of the senior authors (PB, DD).

Data were extracted into a pre-templated charting table, developed iteratively in a piloting stage, by one reviewer and additionally verified by one other. Extracted data included first author, year of study, title, journal, country, study type, level of evidence, number of cases, aims, indications for surgery, mean/median follow-up time, interventions/comparators, outcomes, key findings, and authors’ conclusions. The Oxford Centre for Evidence-Based Medicine (OCEBM) framework was used to categorise studies according to level of evidence. This classification was used to describe the design and hierarchy of the available evidence and was not intended as an assessment of methodological quality or risk of bias. Indications for surgery were calculated as a proportion for each study and charted as whole percentages.

Annual reports published between 2000–2026 from NJRRs in Australia, Canada, Denmark, Finland, Germany, India, Italy, Japan, the Netherlands, New Zealand, Norway, Romania, South Africa, Sweden, Switzerland, the United Kingdom, and the United States were sought. These registries were recently highlighted as those which record data on rTKA and publish their findings in publicly available annual reports [38]. Reports were downloaded to a shared Google drive, screened by two reviewers, and data charted using the same method described above. Consecutive annual reports from the same NJRR were considered serial publications from a single underlying data source rather than independent studies. Where multiple reports presented overlapping or cumulative analyses, only the most recent relevant report was included, with earlier reports additionally retained only where they provided unique historical or supplementary data.

Data from the charted tables were synthesised narratively using a descriptive and thematic approach, structured around five key themes identified from the literature. Included studies were organised into a ‘map of the evidence’, comprising two tables that cross-tabulated the level of evidence against these five themes. The first table mapped all evidence included in the scoping review, whereas the second was restricted to studies in which the cohort was clearly defined as exclusively comprising first-time elective aseptic rTKA procedures.

Ethical approval was not required for this scoping review. This review was informed by the findings of the JLA PSP on the ‘problematic knee replacement’, which incorporated extensive Patient and Public Involvement [6].

## Results

Following deduplication, the searches identified 7,096 research articles and 155 reports from 16 NJRRs. Annual reports from the Indian NJRR could not be located. After application of the eligibility criteria, 116 research articles and six reports from four NJRRs contained relevant data. Supplementary citation checking identified a further five research articles, giving a total of 121 studies and six NJRR reports included in the evidence synthesis. Of the included research articles, 22/121 (18%) had cohorts clearly defined as exclusively comprising first-time elective aseptic rTKA procedures; the remainder included either mixed cohorts or did not clearly define the cohort as such. The PRISMA flowchart is shown in Fig 1. All data extracted from the included studies can be found in Supporting Information File 2.

**Fig 1:**
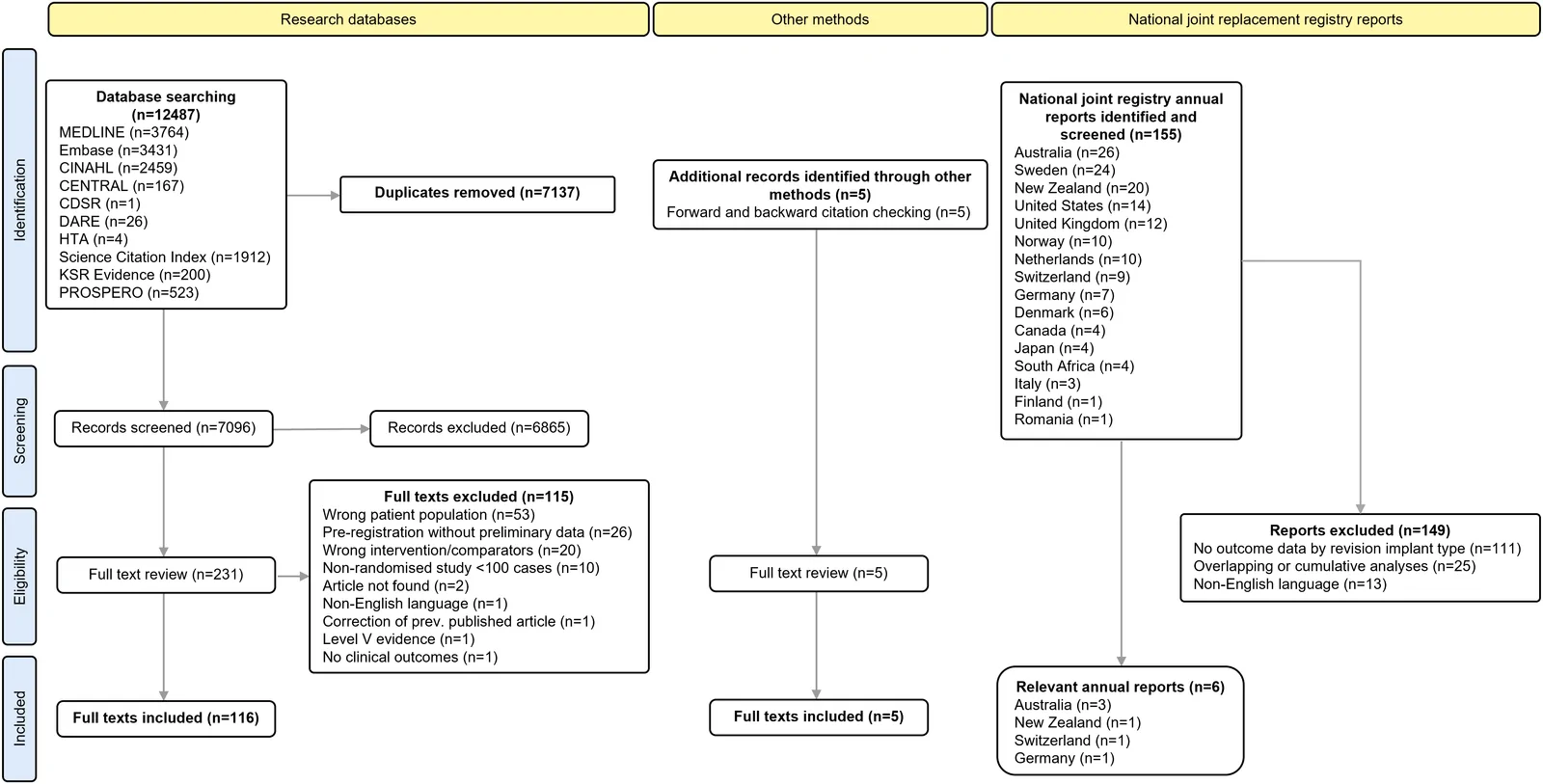
PRISMA flow diagram.

The majority of the included studies were retrospective case series (45/121, 37%) or retrospective comparative studies (41/121, 34%) (Fig 2). The included evidence corresponded to mostly Level IV (48/121 40%), followed by Level III (46/121, 38%), and Level II (27/121, 22%). No Level I evidence was identified. Six articles relating to randomised controlled trials (RCTs) were included, however, three of these report outcomes at consecutive time-points from a single trial conducted between 2008 and 2010 [39–41]. Twenty-one systematic reviews were included, all of which were composed from mostly Level III and IV data, and with marked case-mix heterogeneity [11,14–33].

**Fig 2:**
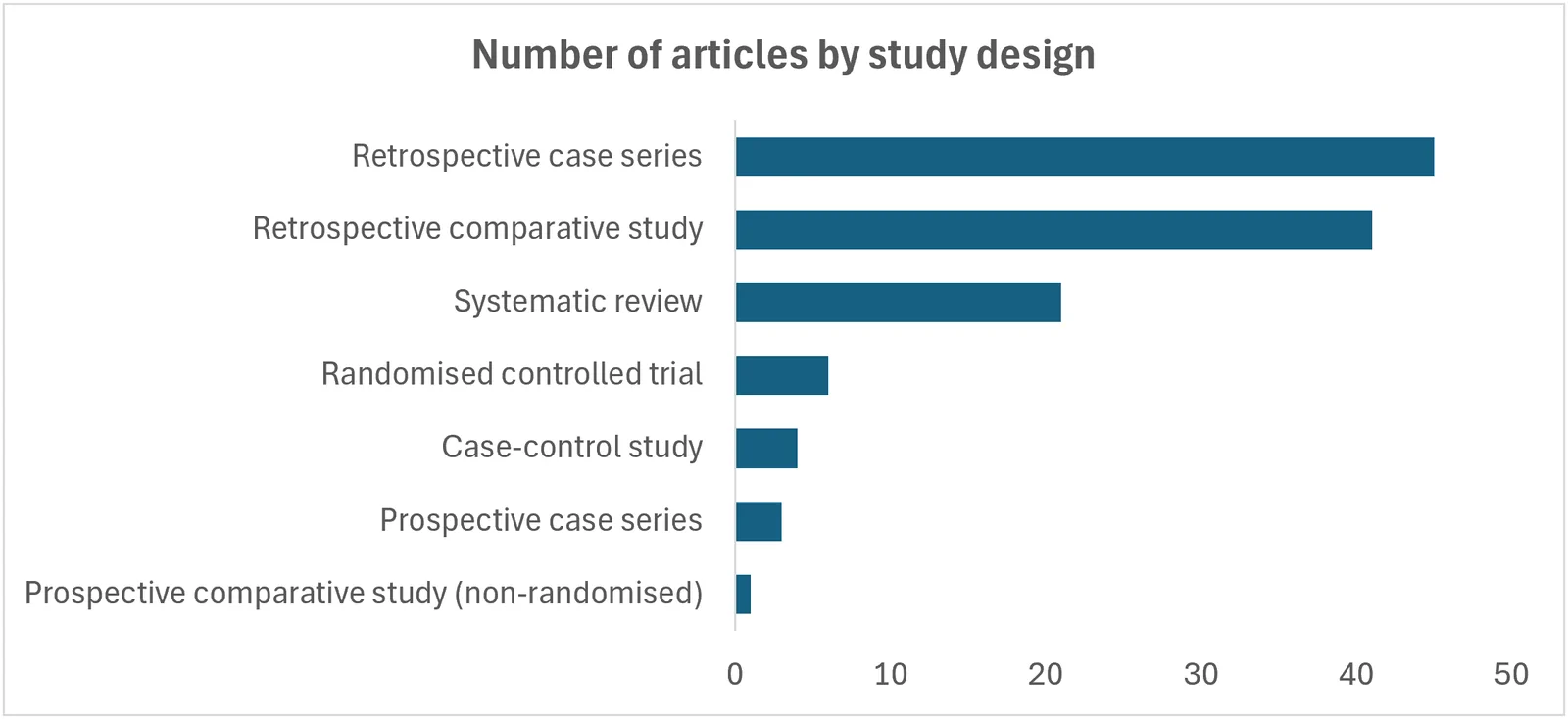
Number of articles by study design.

Approximately half of the included studies were published in the last five years (Fig 3). Approximately half of the studies were conducted in the United States (58/121, 48%) (Fig 4), with most published in the Journal of Arthroplasty (46/121, 38%) (Fig 5). Five studies reported a mean or median follow-up ≥ 10 years [41–45]. A registry-based study from Australia represents the largest individual study included in this scoping review (n = 22238) [46].

**Fig 3:**
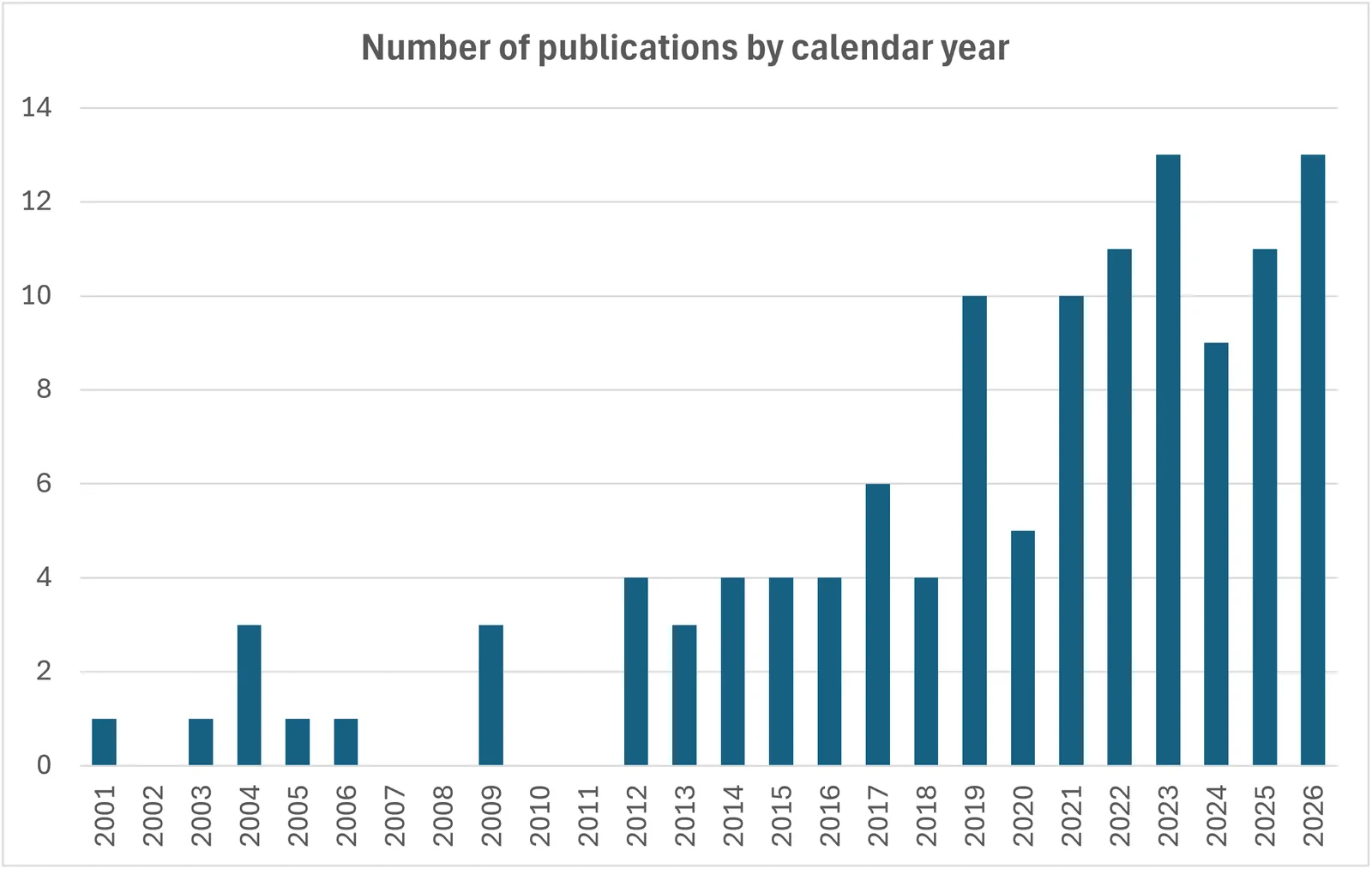
Number of publications by calendar year.

**Fig 4:**
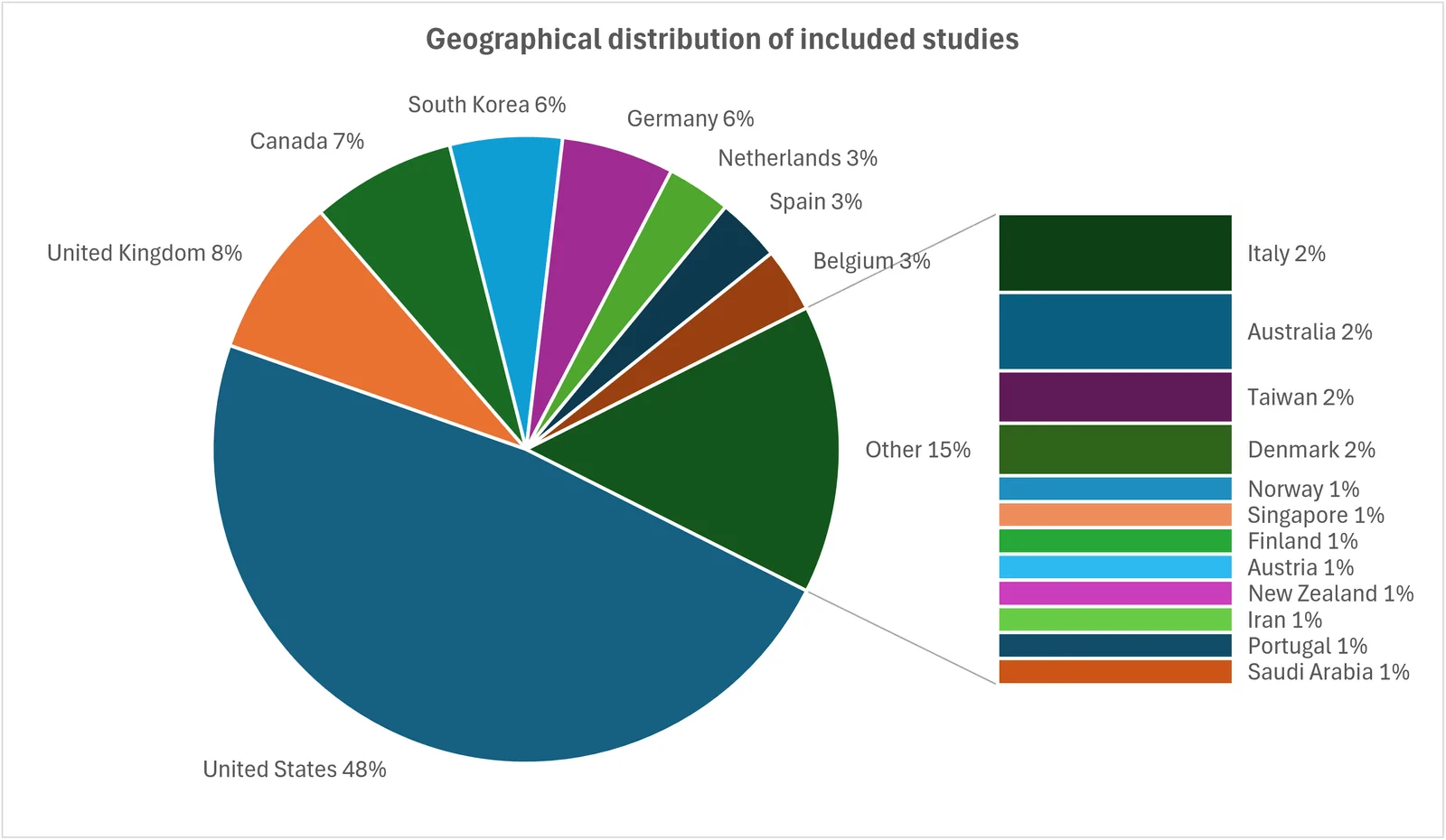
Geographical distribution of included studies.

**Fig 5:**
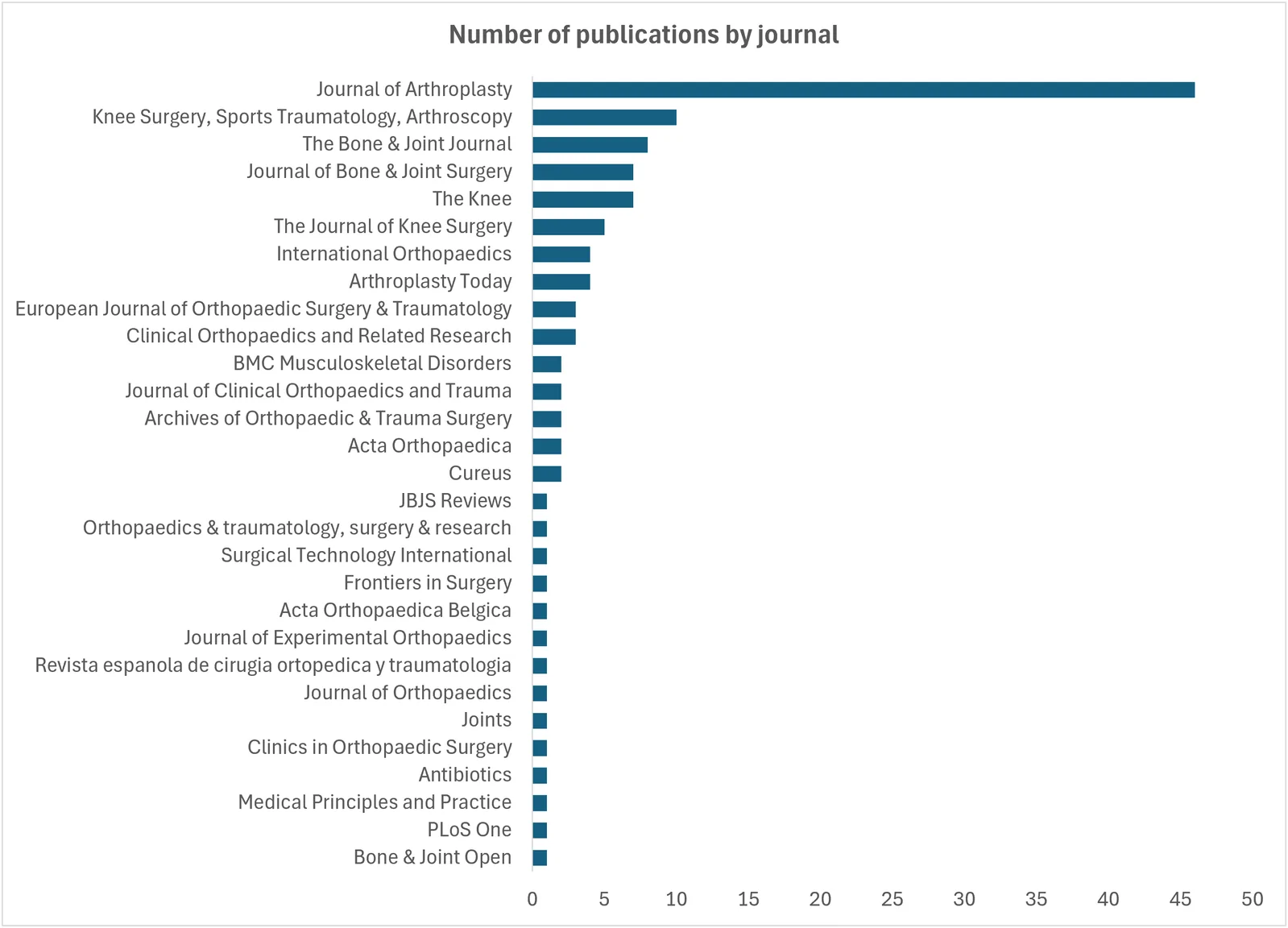
Number of publications by journal.

Registry reports from Australia, New Zealand, Switzerland and Germany contained data relevant to this scoping review [47–52]. Of note, the 2020 annual report from the Australian Orthopaedic Association National Joint Replacement Registry (AOANJRR) contains a focused section on outcome after first revision of a pTKA [47].

Five key themes were identified from the literature: 1) cemented versus hybrid/cementless intramedullary stems; 2) constraint; 3) isolated polyethylene exchange; 4) single- versus full-component revision; and 5) metaphyseal fixation devices. Subsequent synthesis of the results is presented under these headings. A map of the evidence, as defined in the Methods, is provided in Tables 2 and 3.

**Table 2:** Map of all evidence included in the scoping review.

	Level II	Level III	Level IV	Registry data
**Cemented versus hybrid fixation**	[11,17,23,25,26,39–41]	[42,53–61]	[62–69]	
**Constraint**	[24,27,31,70]	[42,57,60,71–81]	[44,45,64,66,69,82–93]	[48]
**Isolated polyethylene exchange**	[30]	[46,74,75,77,94–100]	[83,101–106]	[49–52]
**Single- versus full-component revision**	[21,32,33]	[43,46,57,96,107–113]	[114–116]	[47,48,51]
**Metaphyseal fixation devices**	[14–16,18–20,22,28,29,117]	[10,46,53,59,118–122]	[123–133]	[47,48]
**Other**	[134]	[72,121,122,135–138]	[129,139–143]	

**Table 3:** Map of evidence included in the scoping review, restricted to studies in which the cohort was clearly defined as exclusively comprising first-time elective aseptic rTKA procedures.

	Level II	Level III	Level IV	Registry data
**Cemented versus hybrid fixation**				
**Constraint**		[72,74,81]	[85,86]	[48]
**Isolated polyethylene exchange**		[46,74,94,95,97,98]	[101,104,106]	[52]
**Single- versus full-component revision**		[46,108,109,113]	[115,116]	[48]
**Metaphyseal fixation devices**		[10,46]		[47,48]
**Other**	[134]	[72,138]	[143]	

### Cemented versus hybrid/cementless intramedullary stems

Data from the AOANJRR (n = 5253) highlighted first-time revision of a pTKA performed for aseptic loosening was associated with a significantly lower re-revision rate when an intramedullary stem was used (hazard ratio (HR)=1.59 (95% confidence interval (CI) 1.39, 1.82), p < 0.001) [47]. In the remaining literature, there was no clear consensus with regards to which fixation method of intramedullary stems is optimal. Seven studies supported a hybrid technique [11,23,42,62,63,67,68], five supported the use of cemented stems [53,60,64–66], and 13 studies reported no evidence of a difference in outcomes between the two [17,25,26,39–41,54–57,59,61]. Registry-based studies from Finland (n = 2637, supports cemented fixation) and Norway (n = 1016, supports no difference) reported equivocal findings [57,65]. No studies were identified on this subject with cohorts clearly defined as exclusively comprising first-time elective aseptic rTKA procedures.

Systematic reviews and meta-analyses on this topic are challenging owing to the heterogeneity of studies which are often poor-quality [11,17,23,25,26]. Three articles reporting results from a single randomised study (n = 32) demonstrated no difference in patient-reported outcomes or implant migration, as measured by radiostereometric analysis, at 2, 6.5, and 10 years, respectively [39–41].

Level III evidence was similarly limited. Fiedler et al. reported no difference in re-revision rates when stem type alone was considered, however, significantly higher re-revision rates were noted with the use of offset cementless stems in association with metaphyseal tibial cones (n = 358) [53]. Van Laarhoven et al. report superior survival of fully cemented fixation compared to hybrid fixation with the use of a rotating hinge implant (n = 275) [60]. Although two systematic reviews included in this review favour a hybrid technique [11,23], no original comparative studies favoured this method.

Stem-tip pain has previously been associated with press-fit stems, however, two comparative studies found no difference in the incidence of stem-tip pain between cemented and cementless stems [56,61]. One study reported that a tight diaphyseal fit at the isthmus may minimise the incidence of this phenomenon [63], whilst another demonstrated slotted titanium stems may be favourable over solid, fluted cobalt/chrome stems [62]. Bridger et al. reported that 88.9% of patients experienced improvements in stem pain following non-operative measures [61]. Three systematic reviews explored differences in patient-reported outcomes between cemented and hybrid techniques but did not find any significant differences between the two [11,23,25].

### Constraint

Five studies examined outcomes associated with constraint level in cohorts clearly defined as exclusively comprising first-time elective aseptic rTKA procedures [72,74,81,85,86]. In a large registry-based study from the United Kingdom, Tyas et al. reported that condylar-constrained (CCK) prostheses were associated with the lowest re-revision rates following first-time aseptic rTKA when compared with unconstrained, posterior-stabilised (PS) and hinged implants (n = 12950) [81]. Subgroup analyses demonstrated CCK implants were associated with the lowest re-revision rates in patients revised for aseptic loosening, instability, men, women, patients aged under 55 years, and patients undergoing index rTKA within two years of primary arthroplasty [81]. Recent case series similarly report favourable implant survivorship associated with CCK prostheses [69,92,93].

Further evidence to support the choice of constraint in these patient subgroups was otherwise limited. Registry data from Australia demonstrate an increased risk of re-revision following rTKA for instability associated with the use of PS implants versus fully stabilised prostheses (HR 1.58 (CI 1.14, 2.18), p = 0.005) [48]. Small comparative analyses examining the choice of constraint at rTKA for aseptic loosening demonstrated conflicting results, with Kunze et al. supporting the use of PS implants (n = 148) and Shichman et al. reporting improved outcomes with CCK prostheses (n = 112) [73,79]. One study investigated risk factors for re-revision in patients less than 55 years following aseptic rTKA and found those undergoing revision to a hinged prosthesis experienced higher re-revision rates versus those with PS implants (HR = 3.47, p = 0.05, n = 197) [83].

A recent systematic review and meta-analysis by Ruelos et al. demonstrated patients receiving rotating hinged implants experienced greater improvements in range of motion when compared with non-hinged revision implants in the treatment of arthrofibrosis (p = 0.002, n = 332) [31]. Bingham et al. demonstrated similar results in this patient group but noted higher re-revision rates in patients receiving a rotating hinge construct compared to lesser constrained implants (p = 0.03, n = 102) [71].

Regarding patient-reported outcomes, Turnbull et al. (n = 112) found construct constraint did not affect functional outcome or satisfaction, when adjusting for age, gender, indication for surgery, and several other factors [90]. Other retrospective comparative studies on this topic investigating patient-reported outcomes largely supported this finding [75–77,79,80]. Tyas et al. reported favourable patient-reported outcomes associated with CCK implants in an unadjusted subgroup analysis (n = 2268) [81].

A single randomised study compared the LINK Endo-Model and the Aesculap EnduRo RHK and demonstrated both provided significant improvement in pain and function scores in patients revised for instability (n = 51) [70]. Other Level II evidence was limited, consisting of two systematic reviews comparing CCK and rotating hinge implants, both including mostly small, retrospective studies [24,27].

### Isolated polyethylene exchange (IPE)

The majority of studies evaluating IPE found it was associated with higher re-revision rates compared to component revision [46,74,83,94,95,98,102,104,105]. This was supported by registry data from Australia, New Zealand, Switzerland and Germany [49–52]. The most recent data from the AOANJRR on this subject was provided in the 2024 annual report (n = 28310), demonstrating cumulative 10-year re-revision rates following aseptic rTKA of 25.3% (CI 23.6, 27.0) for IPE versus 18.8% (CI 17.7, 19.9) for full-component revision [52]. In a large systematic review examining IPE for instability (n = 4269), Stroobant et al. reported poor outcomes, with recurrent instability and re-revision occurring in 6.5%−18.5% of cases [30].

There were limited studies supporting IPE, however, some suggested favourable results may be obtained in those revised for polyethylene wear [103,105] or stiffness [97,99]. Parish et al. recently reported favourable patient-reported outcomes when IPE was used for the management of global instability with well-fixed components [100]. Data from Germany suggested re-revision rates may be improved when secondary patella resurfacing is also undertaken [106]. Another study demonstrated results between IPE and full-component revision were comparable and suggested polyethylene sterilisation had the greatest influence on re-revision rates [96].

### Single- versus full-component revision

The majority of individual studies supported the role of single-component revision in selected cases [43,96,107–110,114,115]. All these studies included fewer than 300 patients. Three systematic reviews compared single- versus full-component revision; one reported single-component revision was associated with higher re-revision rates, another reported comparable re-revision rates, and the third reported that the poor quality of the included studies precluded sound conclusions [21,32,33]. Howard et al. (n = 262) reported a median follow-up time of 10 and eight years, respectively, in a comparison of single- and full-component revisions [43], however, all other studies had mean/median follow-up less than seven years.

Compared with isolated tibial component revision, isolated femoral component revision was associated with an increased risk of re-revision in a registry-based study from New Zealand (HR 1.70, p = 0.042, n = 1720) [116]. In a registry-based study from Norway, single-component revision had a higher risk of re-revision than exchange of the whole prosthesis (relative risk = 1.7, p = 0.02, n = 1016) [57]. Registry data from Australia and Switzerland also demonstrated higher re-revision rates associated with single component revision [48,144]. Following rTKA for aseptic loosening, the most recent data from the AOANJRR indicate 10-year re-revision rates of 22.3% (CI 20.4, 24.4) for isolated tibial component revision, 29.4% (CI 25.7, 33.4) for isolated femoral component revision and 18.9% (CI 17.5, 20.3) for full component revision (n = 7504) [48]. Beyond 1.5 years, the difference in re-revision rates between single- and full-component revision following all first-time aseptic rTKA is not statistically significant after adjusting for age and gender (HR = 1.11 (0.98, 1.27), p = 0.118) [52].

Few studies reported alternative outcome measures. Lindberg et al. reported a trend towards increased 90-day reoperation rates associated with single-component revision, however, other studies reported similar rates of postoperative surgical complications between single- versus full-component revisions [43,107,112,113]. Regarding single-component revision, Lin et al. demonstrated shorter operative times (p = 0.003), supported by a systematic review by Sheth et al., and shorter hospital length of stay (p = 0.002) [32,108]. A systematic review by Hajiaghajani et al. reported similar patient-reported outcomes between single- versus full-component rTKA procedures [33].

### Metaphyseal fixation devices

Nine systematic reviews investigating the role of metaphyseal fixation devices were identified – three summarised the outcomes of metaphyseal sleeves [15,20,28]; two compared outcomes between bone grafting and metaphyseal fixation devices [14,16]; and four compared outcomes between metaphyseal sleeves and metaphyseal cones [18,19,22,29]. Three reviews reported higher reoperation rates associated with metaphyseal cones compared with metaphyseal sleeves [18,22,29], however, applying the OCEBM framework, all included only Level IV evidence. None of the above systematic reviews included comparative studies investigating outcomes between metaphyseal cones and sleeves. All Level III evidence on this topic was published within the last four years (Table 2).

Only two studies investigated outcomes associated with the use of metaphyseal fixation devices in a clearly defined cohort comprising exclusively first-time elective aseptic rTKA procedures [10,46]. Hoskins et al. reported that, among patients undergoing full-component revision with tibial and femoral stems, constructs without metaphyseal fixation devices were associated with a higher risk of all-cause re-revision than those incorporating metaphyseal sleeves or cones, although this difference did not reach statistical significance (HR 1.19, 95% CI 0.95 to 1.48; p = 0.13) [46]. Hinman et al. similarly demonstrated no statistically significant difference in aseptic re-revision rates between rTKA with stems alone versus rTKA with stems and metaphyseal fixation devices (n = 2882) (HR = 0.76, CI 0.42 to 1.37) [10]. After adjusting for age and gender, data from the AOANJRR (n = 5253) demonstrated rTKA for aseptic loosening was associated with lower re-revision rates when a metaphyseal sleeve was used versus revision without sleeves (HR = 1.37, CI 1.08–1.72, p = 0.008), whilst the use of metaphyseal cones was not found to affect re-revision rates (HR = 0.82, CI 0.38, 1.70, p = 0.573) [47]. Analysis of the latter in this report included only 79 revisions with a metaphyseal cone.

Bloch et al. and Agarwal et al., reported favourable outcomes of metaphyseal sleeves at a mean follow-up of 7.6 and 8.0 years, respectively [123,125]. All other included studies reporting on metaphyseal fixation devices reported results in the short- to mid-term.

A single randomised study investigating the role of metaphyseal fixation devices was identified [117]. This study investigated adaptive bone remodelling of the proximal tibia and tibial shaft after rTKA with or without a metaphyseal cone and demonstrated no significant differences in bone-mineral density changes or KSS between the two groups (p = 0.36, n = 40).

The incidence of intraoperative periprosthetic fracture in association with the use of metaphyseal fixation devices, particularly metaphyseal sleeves, is commonly discussed in the literature [15,28,29,126,127,132,133]. The incidence of this complication in association with implantation of metaphyseal sleeves was found to be between 1.0% and 3.7%, with Bonanzinga et al. reporting that most occurrences were undisplaced fractures not requiring further surgical treatment [15,28,29]. One study reported low incidence of intraoperative fractures (1%) associated with the use of flexible metaphyseal cones [133].

### Other studies

Two studies demonstrated the use of antibiotic-loaded bone cement in aseptic rTKA was associated with a lower risk of prosthetic joint infection post-operatively [72,134]. Two studies supported retention of a well-positioned, stable patella component in aseptic tibiofemoral rTKA [141,143]. Two studies reported the outcomes of specific revision knee systems, with a variety of reconstructive techniques employed [135,139]. Bugbee et al. supported the use of revision knee systems at rTKA as opposed to using primary implants [136]. Five studies examined reconstructive techniques in Zone 1; Berend et al. reported on the outcomes of rTKA using cement and screws [137], whilst four studies reported outcomes associated with the use of metal augments [121,122,138,142]. Two studies reported favourable mid-term survivorship and clinical outcomes for mobile-bearing tibial trays [129,140].

## Discussion

To our knowledge, this is the first scoping review to map the evidence for the implants used at elective aseptic revision of a pTKA. The findings demonstrate that the available evidence is severely limited and consists predominantly of retrospective case series and comparative studies, with few reporting long-term outcomes. Studies examining implant selection in clearly defined cohorts undergoing first-time elective aseptic rTKA were also uncommon; no studies investigated outcomes associated with intramedullary stem design in this population, and only two examined the use of metaphyseal fixation devices in this setting.

The optimal fixation method for intramedullary stems remains a subject of ongoing debate, with no clear consensus established. Some authors suggest cemented stems allow greater flexibility in component positioning and provide greater initial implant stability [53]. Others suggest cementless stems with offset couplers are associated with superior anatomic alignment and may also avoid the challenges of cement removal at future re-revision procedures [25,67]. The increased construct complexity associated with press-fit stems and offset couplers might be assumed to increase implant costs, however, the comparative health economics of cemented and cementless stems are not well understood. Our review highlights the paucity of evidence available to support the choice of intramedullary stem design at first-time elective aseptic rTKA. Future studies should compare long term implant survivorship in this patient population and explore whether specific scenarios favour the use of one technique over the other.

The literature commonly supports using the lowest level of constraint required to achieve adequate joint stability, while considering the potential implications of greater constraint for long-term implant survivorship [12,13]. However, recent data from the National Joint Registry suggest CCK prostheses are not associated with compromised implant survival following first-time aseptic rTKA [81]. Nevertheless, prospective evidence is lacking and longer-term follow-up is required. Future research should compare constraint levels within clearly defined and clinically homogeneous revision scenarios, particularly where equipoise may exist between PS and CCK implants. Such studies should incorporate detailed measures of bone loss, ligament competence, intraoperative stability and the broader reconstructive strategy to reduce confounding by indication. Longer-term implant surveillance should be complemented by robust patient-reported measures to establish whether greater constraint influences functional outcomes and perceived knee stability in addition to re-revision risk.

The evidence suggests that whilst IPE may be associated with less morbidity in the short-term, longer-term outcomes depend heavily on the indication for revision and appropriate patient selection. Where malalignment or component malposition are present, the literature generally describes full-component revision as the preferred approach. Although we were unable to identify evidence above Level III, the findings across studies were relatively consistent, suggesting that the principal areas of uncertainty may now relate more to patient selection than to whether IPE has a role in selected cases.

The available registry-data presented in this review suggests a tendency towards increased re-revision rates following single- versus full-component revision. However, differences in other outcome measures such as operative times, surgical complications and length of stay are less well-established. Future research might look to identify which patients are appropriate to be considered for single-component revision versus those in which it is ill-advised. Although conducting RCTs in rTKA presents challenges, this is one area in which an RCT might be considered if there were to be sufficient equipoise and further feasibility work supported the potential for a trial.

Registry data from the AOANJRR report favourable outcomes for metaphyseal sleeves at first-time revision for aseptic loosening, however, the evidence-base for metaphyseal cones is less developed [47]. Comparative evidence is otherwise yet to demonstrate statistically significant improvements in implant survivorship associated with the use of metaphyseal fixation devices at first-time aseptic rTKA. In the study by Hinman et al., the proportion of rTKA procedures utilising metaphyseal fixation devices increased from 7.5% in 2008 to 42.8% in 2022, without an observable effect on re-revision rates [10]. Given the additional implant cost associated with metaphyseal fixation devices, further evidence regarding their comparative clinical effectiveness and cost-effectiveness would be valuable, particularly in publicly funded healthcare systems. Further systematic reviews are unlikely to substantially advance our understanding on this subject until shortfalls in the primary evidence base are addressed. The extent to which the use of these devices is influenced by non-clinical factors, such as surgeon training, local procurement practices and industry relationships, is also not well understood.

### Limitations

This scoping review has several limitations. The eligibility criteria may have resulted in the exclusion of some relevant published evidence. For example, studies were excluded if more than 30% of the cohort underwent revision for prosthetic joint infection or periprosthetic fracture, even if subgroup analyses stratified results by revision indication. Conversely, while patients undergoing first-time aseptic rTKA were the population of interest, some included studies include patients revised for non-elective indications. Alternative approaches to the design of the eligibility criteria were considered by the authorship team *a priori.* The 30% threshold was chosen as a pragmatic balance between maintaining relevance to the population of interest and avoiding the exclusion of otherwise informative studies. The same threshold has been used in previous systematic reviews on this subject [145]. Studies focused on reconstructive techniques and implant selection in complex rTKA are unlikely to have been included in this review. Whilst it might be argued that reconstructive techniques used in more complex rTKA scenarios are likely to be equally or perhaps more successful in ‘lesser’ revision knee procedures, we have attempted to direct the reader to evidence supporting targeted and appropriate implant selection at first-time elective aseptic rTKA. Finally, although the OCEBM framework was used to describe the level of evidence, it does not constitute a formal assessment of methodological quality or risk of bias, and this should be carefully considered when drawing conclusions from the evidence mapped in this review.

## Conclusions

In conclusion, the literature to support implant selection at elective aseptic revision of a primary total knee replacement is dominated by retrospective observational studies, with limited prospective or randomised comparative evidence. This review provides a map of the current evidence to support decision-making in this setting. Where feasible, future comparative studies should reduce clinical heterogeneity by focusing on clearly defined cohorts of first-time elective aseptic rTKA procedures. Future research should compare long-term outcomes between cemented versus hybrid/cementless stems; constraint levels; and rTKA with or without metaphyseal cones or sleeves. Whilst NJRR annual reports rarely provide sufficient granularity, registry datasets may represent the greatest potential platform on which to answer some of these research questions.

## Supporting information

S1 File MEDLINE search strategy. (PDF)

S2 File All extracted data from the included studies. (DOCX)

S3 File PRISMA-ScR checklist. (PDF)
