# Supplementary material for: Implant selection at elective aseptic revision of a primary total knee replacement: A scoping review and map of the evidence
Source: PLoS One. 2026 Oct 21;21(10):e0361040. doi: 10.1371/journal.pone.0361040 (PMC13686483; doi:10.1371/journal.pone.0361040)
Supplement: Supplementary file 1 [file pone.0361040.s001.pdf]

S1 File. MEDLINE Search Strategy.

Ovid MEDLINE(R) ALL <1946 to May 16, 2025>
Search date and time: 19/5/25 0950; Records retrieved: 3370

1	Arthroplasty, Replacement, Knee/	35715
2	Reoperation/	99911
3	1 and 2	5262
4	((revis* or reoperat* or re operat*) and knee and (arthroplast* or replac* or resurfac*)).ti,kf.	3299
5	((revis* or reoperat* or re operat*) adj5 knee adj5 (arthroplast* or replac* or resurfac*)).ab.	2995
6	4 or 5	4711
7	3 or 6	7599
8	Knee Prosthesis/	14698
9	Prosthesis Design/	64376
10	(knee adj2 (prosthes?s or implant?)).ti,ab,kf.	4545
11	((femur or femoral) adj2 (prosthes?s or implant?)).ti,ab,kf.	2842
12	((tibia or tibial or tibiofemoral or tibio-femoral) adj2 (prosthes?s or implant?)).ti,ab,kf.	955
13	((patella or patellofemoral or patello-femoral) adj2 (prosthes?s or implant?)).ti,ab,kf.	142
14	8 or 9 or 10 or 11 or 12 or 13	78324
15	7 and 14	3546
16	(stem* or sleeve? or cone? or augment?).ti,ab,kf.	729840
17	(cement* or uncemented or press fit or pressfit).ti,ab,kf.	77116
18	(constrain* or semiconstrain* or unconstrain*).ti,ab,kf.	216211
19	(cruciate adj2 (retain* or substitut*)).ti,ab,kf.	1522
20	((posterior* or total*) adj2 stabili*).ti,ab,kf.	4702
21	((rotating or fixed) adj2 hinge*).ti,ab,kf.	416
22	(hinge* adj2 (implant* or prosthes*)).ti,ab,kf.	548
23	((hybrid or zone* or zonal or implant* or prosthes*) adj2 fixation*).ti,ab,kf.	3336
24	fixed bearing*.ti,ab,kf.	1084
25	mobile bearing*.ti,ab,kf.	1356
26	implant system*.ti,ab,kf.	3436
27	(polyethylene adj2 (insert? or liner*)).ti,ab,kf.	1913
28	or/16-27	1020561
29	7 and 28	2700
30	15 or 29	4298
31	exp animals/ not humans/	5346762
32	30 not 31	4293
33	limit 32 to yr="2000 -Current"	3996
34	limit 33 to english language	3801
35	case reports.pt.	2481707
36	(case adj (report or reports or study or studies)).ti.	446702
37	35 or 36	2589921
38	34 not 37	3426

39	(comment or editorial or letter).pt.	2326793
40	38 not 39	3370
