# Supplementary material for: Implant selection at elective aseptic revision of a primary total knee replacement: A scoping review and map of the evidence
Source: PLoS One. 2026 Oct 21;21(10):e0361040. doi: 10.1371/journal.pone.0361040 (PMC13686483; doi:10.1371/journal.pone.0361040)
Supplement: Supplementary file 3 [file pone.0361040.s002.docx]

**S2 File. All extracted data from the included studies.**

| **First author** | **Year** | **Title** | **Journal** | **Country** | **Study type** | **Level of evidence** | **No. cases** | **Aims** | **Indications** | **Follow up (Years)** | **Interventions** | **Outcomes** | **Key findings** | **Authors conclusions** | **Topic(s)** |
| --- | --- | --- | --- | --- | --- | --- | --- | --- | --- | --- | --- | --- | --- | --- | --- |
| Agarwal | 2018 | Metaphyseal sleeves in revision total knee arthroplasty: Minimum seven-year follow-up study | The Knee | United Kingdom | Case series | IV | 104 | To report 7 year outcomes of metaphyseal sleeves | Aseptic loosening 41%, Instability 12%, Undiagnosed pain 7%, Stiffness 10%, Infection 30%, Failed unicompartmental 1% | 8 | rTKA with metaphyseal sleeves | Re-revision, OKS | OKS 28.5 (in those without re-revisions); 23/104 (22.1%) re-revisions | At mid-term follow-up, metaphyseal sleeves demonstrate durable clinical results and radiographic fixation | Zone 2 fixation devices |
| Agarwal | 2013 | Metal metaphyseal sleeves in revision total knee replacement | The Bone & Joint Journal | United Kingdom | Case series | IV | 104 | To report early results of metaphyseal sleeves | Aseptic loosening 41%, Instability 12%, Undiagnosed pain 7%, Stiffness 10%, Infection 30%, Failed unicompartmental 1% | 3.6 | rTKA with metaphyseal sleeves | Re-revision, ROM, OKS | OKS improved 23 to 32; 2/104 (1.9%) re-revisions | These early results encourage us to continue with the technique and monitor the outcomes in the long term | Zone 2 fixation devices |
| Alexander | 2023 | Isolated Tibial Polyethylene Insert Exchange After Total Knee Arthroplasty for Treatment of Instability and/or Polyethylene Wear | Surgical Technology International | United States | Case series | IV | 390 | To evaluate survival and outcomes of isolated polyethylene exchange for instability/polyethylene wear | Instability/Polyethylene wear 100% | 5.9 | Isolated polyethylene exchange | Re-revision, ROM, KSC | KSC improved 55 to 76; 30/390 (7.7%) re-revision; 10-yr survival 84.7% | More studies should be conducted that assess the differences in outcomes of ITPIE as compared to full knee revision | Isolated polyethylene exchange |
| Alhifzi | 2025 | Outcomes and Challenges of Optimal Fixation Selection for Metaphyseal Fixation Techniques in Revision Knee Arthroplasty According to Bone Defects: A Systematic Review | Cureus | Saudi Arabia | Systematic review | II | 544 | To compare clinical and radiographic outcomes and associated complications of metaphyseal sleeves and cones across varying defect severities | Not clearly defined; heterogeneous | Mean range 3.3-9.0 | rTKA with metaphyseal sleeves or cones | Implant survivorship, Re-revision, KSS, VAS, WOMAC, ROM, Radiographic assessment, Periprosthetic fracture | Metaphyseal sleeves demonstrated a 100% survival rate over five years across multiple studies, while metaphyseal cones showed a survivorship rate of 91 91%. Both techniques demonstrated satisfactory functional recovery. Sleeves were associated with a higher incidence of intraoperative fractures (3.7%), whereas cones required more re-revision surgeries (5.33%), often due to issues with cement fixation. | Both metaphyseal sleeves and cones provided effective fixation in rTKA. Neither method proved universally superior; the choice should be tailored to the severity of the defect and individual patient factors | Zone 2 fixation devices |
| Apinyankul | 2023 | Isolated Versus Full Component Revision in Total Knee Arthroplasty for Aseptic Loosening | Journal of Arthroplasty | United States | Retrospective comparative study | III | 238 | To examine outcomes of isolated vs full component revision for aseptic loosening | Aseptic loosening 100% | 5.1 | Single component vs full component revision | Re-revision, Multiple PROMs, Surgical complications | No difference in re-revision/clinical scores | Isolated and full component revision TKA for aseptic loosening does not differ with respect to prosthesis failures, complications, and clinical results at 5 years. | Single- vs full-component revision |
| Bae | 2013 | Long-term survival rate of implants and modes of failure after revision total knee arthroplasty by a single surgeon | Journal of Arthroplasty | South Korea | Case series | IV | 224 | To evaluate long term results after rTKA by single surgeon | Polyethylene wear 53%, Aseptic loosening 28%, Infection 10%, Other 9% | 8.1 | rTKA with DePuy PFC, all posterior stabilised inserts | Re-revision, Implant survival, KSS, ROM | 20/224 (8.9%) re-revisions; 10-yr survival 86.1% | The long-term survival rate of revision TKA was satisfactory, but careful attention is necessary to detect the late failure. | Constraint |
| Barrack | 2004 | The effect of stem design on end-of-stem pain in revision total knee arthroplasty | Journal of Arthroplasty | United States | Case series | IV | 143 | To determine whether the incidence of end-of-stem pain was influenced by stem design | Infection excluded, otherwise not clearly defined | 5 | Solid, fluted CoCr stem vs slotted Ti stem | Re-revision, KSS, UCLA, Patient pain maps | Higher incidence of end-of-stem pain associated with solid CoCr stems | A slotted titanium stem appears to be a desirable design feature for revision TKA. | Cemented vs hybrid fixation |
| Beckmann | 2015 | Treatment of severe bone defects during revision total knee arthroplasty with structural allografts and porous metal cones-a systematic review | Journal of Arthroplasty | Germany | Systematic review | II | 699 | To determine if TM cones vs structural allografts improve re-revision rates in patients with AORI 2 and 3 defects | Not defined; heterogeneous | Mean range 0.9-8.9 | Structural allograft vs trabecular metal cones | All cause re-revision, infection rate, aseptic loosening rate | All included studies were case series <100cases; Metaregression revealed an odds ratio for aseptic loosening of 0.263(CI 0.085 to 0.816,P=0.021) in favour of cones | The meta-regression results should not be considered conclusive but rather strongly indicative of improved results with the use of TM cones. | Zone 2 fixation devices |
| Berend | 2015 | Use of screws and cement in revision TKA with primary or revision specific prosthesis with up to 17 years followup | Journal of Arthroplasty | United States | Retrospective comparative study | III | 609 | To examine the efficacy of cement reinforced with screws | Not defined | 7.4 | Cemented rTKA with primary/revision prosthesis with/wiithout screw augmentation | Re-revision, Implant survival, Knee/pain/flexion scores | KM survivorship; revision prosthesis with screws=0.9859, revision prosthesis without screws=0.9848, primary prosthesis with screws=0.9118, primary prosthesis without screws=0.9424 | Use of revision prosthesis along with screws and cement to correct largely defective revision TKAs is highly recommended | Other- Zone 1 augmentation |
| Bingham | 2019 | Rotating-Hinge Revision Total Knee Arthroplasty for Treatment of Severe Arthrofibrosis | Journal of Arthroplasty | United States | Retrospective comparative study | III | 102 | To report arc of motion, Knee Society scores, and implant survivorship in patients with arthrofibrosis revised with an RH | Arthrofibrosis 100% | 6 | rTKA with vs without rotating hinge | Re-revision, Re-operation, KSS, ROM | ROM increased 20deg (74-94) in the RH group vs 12deg (87-99) in the nonRH group (p=0.048). Survivorship free of any revision at 10 years was 54% in the RH group and 90% in the non-RH group (p=0.03). | Patients with arthrofibrosis revised to an RH had a 20deg improvement in ROM and MUA was half as common. However, there was a higher risk of re-revision in the RH group. | Constraint |
| Bini | 2016 | Antibiotic cement was associated with half the risk of re-revision in 1,154 aseptic revision total knee arthroplasties | Acta Orthopaedica | United States | Retrospective comparative study | III | 1154 | To determine the incidence of re-revision following aseptic revision TKA, the causes of failure, and any patient-, surgeon-, hospital-, or procedure-related risk factors that affected the outcome | Aseptic indications 100%; infection excluded | Not stated | rTKA. Re-revision risk factors evaluated; procedure variables included the use of antibiotic-loaded bone cement and the use of hinged prosthesis. | Re-revision | Lower risk re-revision with antibiotic loaded cement (hazard ratio (HR) = 0.5, 95% CI: 0.3-0.9). No association between re-revision and use of hinged prosthesis. | The use of antibiotic-loaded cement, higher age, and higher BMI were associated with lower risk of further revision whereas a higher degree of surgeon experience was associated with higher risk | Cemented vs hybrid fixation; Constraint |
| Bishi | 2026 | Rotating Hinge Knee Versus Constrained Condylar Knee Revision: A Single Centre, Retrospective Comparative Study | Cureus | United Kingdom | Retrospective comparative study | III | 108 | To compare patient-reported outcome measures (PROMs), complication rates and survivorship of RHK and CCK used in revision arthroplasty at a single, high-volume elective orthopaedic centre | Aseptic loosening 42%, Infection 19%, Instability 13%, Polyethylene wear 8%, Unknown 7%, Pain 5%, Other 6% | Not stated | rTKA with rotating hinge knee versus condylar constrained revision knee system | Implant survivorship, EQ5D, Major postoperative surgical complications | There was no statistical significant difference between RHK and CCK in implant survival at two to 12 years of follow up. There was no statistical significant difference in the PROMs scores and complication rates of the two implants. | Both the RHK and CCK remain viable options in revision arthroplasty; the implant survival and complication rates were comparable | Constraint |
| Bloch | 2020 | Metaphyseal Sleeves in Revision Total Knee Arthroplasty Provide Reliable Fixation and Excellent Medium to Long-Term Implant Survivorship | Journal of Arthroplasty | United Kingdom | Case series | IV | 319 | To report implant survivorship and radiographic outcome of 319 sleeves used in rTKA | Aseptic loosening 50%, Infection 22%, Instability 21%, Stiffness 4%, Malalignment 2%, Fracture 1% | 7.6 | rTKA with metaphyseal sleeves | Re-revision, Radiographic assessment | Implant survivorship was 99.1% at 3 years, 98.7% at 5 years, and 97.8% at 10 years. No metaphyseal sleeve was revised for aseptic loosening. | Use of a metaphyseal sleeve in revision TKA is associated with excellent survivorship and radiographic outcome in the medium to long term | Zone 2 fixation devices |
| Boelch | 2018 | Revision knee arthroplasty with rotating hinge systems in patients with gross ligament instability | International Orthopaedics | Germany | Randomised controlled trial | II | 51 | To compare the clinical and radiographic outcomes after rTKA for instability with two rotating hinge knee prostheses | Instability 100% | 1 | LINK Endo-Model vs Aesculap EnduRo RHK | Re-revision, Multiple PROMs, ROM, radiographic assessment | Except for the patient function Knee Society Score and the Physical Health Component Summary Score in the EnduRo group, all clinical scores improved post-operatively. In the EnduRo group 2 patients (8%) and in the Endo-Model group 1 patient (3.8%) had to be revised for infection. | Both prosthetic designs provide significant improvement in pain and function scores after TKA revision for gross instability | Constraint |
| Bonanzinga | 2019 | Are Metaphyseal Sleeves a Viable Option to Treat Bone Defect during Revision Total Knee Arthroplasty? A Systematic Review | Joints | Italy | Systematic review | II | 928 | To summarise the clinical and radiographic outcomes of metaphyseal sleeves in rTKA | Not defined; heterogeneous | 3.8 | rTKA with metaphyseal sleeves | Re-revision, Intraoperative fracture, Multiple PROMs | 36 septic re-revisions of the prosthetic components (4%) The rate of aseptic re-revisions was 27/928 (3%). Clinical outcomes improved. | Metaphyseal sleeves demonstrate high radiographic signs of osteointegration, low septic loosening rate, low intraoperative fracture rate,and a good-to-excellent clinical outcome | Zone 2 fixation devices |
| Bridger | 2026 | Tip-of-Stem Pain Is a Rare Complication Following Revision Total Knee Arthroplasty at a Mean 6.9-Year Follow-Up | Journal of Arthroplasty | Canada | Case-control study | III | 910 | To evaluate the incidence of tip-of-stem pain and its associations with clinical and radiographic outcomes in rTKA | Not defined | 6.9 | rTKA with tibial and/or femoral stems | Tip-of-stem pain, Radiographic assessment (Stem geometry), Re-revision | The incidence of tip-of-stem pain was 3.0%. Longer tibial stems were associated with stem pain, as 100% of patients who had tibial stem pain had a tibial stem length of 100 mm or more (P = 0.004). Overall, 88.9% of patients experienced improvements in stem pain following nonoperative measures, with 59.3% experiencing complete resolution | Despite a low incidence, understanding certain radiographic variables associated with tip-of-stem pain may guide surgeons in the most appropriate stem length, diameter, and design | Cemented vs hybrid fixation |
| Bugbee | 2001 | Does implant selection affect outcome of revision knee arthroplasty? | Journal of Arthroplasty | United States | Retrospective comparative study | III | 139 | To compare clinical outcomes between the use of primary vs revision implant systems in rTKA | Aseptic loosening 39%, Instability 15%, Polyethylene wear 11%, Osteolysis 7%, Failed patella component 6%, Infection 5%, Fracture 6%, Other 8% | 7 | rTKA with primary implants (group 1), modified primary implants (group 2) or revision implants (group 3) | Re-revision, KSS | Re-revision rates= 26% for group 1, 14% for group 2, and 6% for group 3 (p<0.05) | Revision implants exhibited superior performance and durability despite their use in more difficult reconstructions. The improved longevity of revision implants justifies the evolution of modular revision components. | Other- primary vs revision implants |
| Byttebier | 2022 | Comparison of Different Strategies in Revision Arthroplasty of the Knee with Severe Bone Loss: A Systematic Review and Meta-Analysis of Clinical Outcomes | Journal of Arthroplasty | Belgium | Systematic review | II | 4391 | To evaluate the midterm clinical and survivorship results of porous cones and porous-coated sleeves compared to morselized and structural grafts | Not defined; heterogeneous | Mean range 2.0-10.5 | Impaction grafts, structural grafts, sleeves, cones | Implant survivorship, Multiple PROMs | Logistic regression showed non-significant odds ratio (OR) at 10 years of 0.91 (95% confidence interval CI 0.699-1.192, p=0.49) for failure comparing all porous implants with all grafting procedures | Porous implants are effective in tackling metaphyseal bone defects showing good survivorship outcome at midterm follow-up. In younger patients with less constrained prosthetic implants, surgeons might still consider the use of grafts without risking worse outcomes. | Zone 2 fixation devices |
| Chalmers | 2021 | High Rate of Re-Revision in Patients Less Than 55 Years of Age Undergoing Aseptic Revision Total Knee Arthroplasty | Journal of Arthroplasty | United States | Case series | IV | 197 | To characterise the re-revision-free survival and risk factors for re-revision in patients less than 55 years who underwent aseptic revision TKA | Instability 29%, Stiffness 26%, Aseptic loosening 24%, Wear/Osteolysis 9%, Patella complications 6%, Polyethylene fracture/dissociation 2%, Other 2% | 5 | rTKA. Re-revision risk factors evaluated incl. isolated polyethylene exhange vs component revision and constraint | Re-operation, Re-revision | Isolated polyethylene exchange (HR=3.0, p=0.004), and a hinged prosthesis (HR=3.47, p=0.05) were significant risk factors for lower revision-free survival | Patients with one or more prior revision (HR=2.78), rotating hinge components (HR=3.47), or undergoing isolated polyethylene exchanges (HR=3.47) had significantly lower re-revision-free survival. | Isolated polyethylene exchange; Constraint |
| Chalmers | 2021 | Early Survivorship of Newly Designed Highly Porous Metaphyseal Tibial Cones in Revision Total Knee Arthroplasty | Arthroplasty Today | United States | Case series | IV | 163 | To analyse the early survivorship in patients undergoing rTKA with a newly designed highly porous metaphyseal cone | Aseptic loosening 46%, Infection 28%, Instability 15%, Other 11% | 2.5 | rTKA with metaphyseal cone | Implant survivorship, Radiographic assessment, Periprosthetic fracture | Survivorship free from re-revision for aseptic loosening, any nonmodular revision, and any reoperation was 100%, 96%, and 86% at 2 years, respectively | In a complex revision TKA cohort with a new highly porous tibial cone, in which most patients received hybrid stem fixation and nonlinked and linked constraint, there was 100% survival free from re-revision for aseptic loosening at 2 years. Longer term follow-up is required. | Zone 2 fixation devices |
| Chalmers | 2019 | Contemporary Revision Total Knee Arthroplasty in Patients Younger Than 50 Years: 1 in 3 Risk of Re-Revision by 10 Years | Journal of Arthroplasty | United States | Case series | IV | 135 | To determine risk factors for failure in patients younger than 50 years undergoing rTKA | Instability 47%, Aseptic loosening 29%, Arthrofibrosis 14%, Other 10% | 7 | rTKA. Re-revision risk factors evaluated incl. isolated polyethylene exhange vs component revision | Re-revision | Increased risk of re-revision for instability associated with isolated polyethylene exchange (insignficant); HR= 3.47 (CI 0.93-12.78), p=0.06 | Patients <50 years undergoing contemporary aseptic revision TKA had a 1 in 3 risk of re-revision. Patients specifically revised for instability or had prior TKA revisions had the highest risk of re-revision at 10 years. | Isolated polyethylene exchange |
| Chen | 2023 | Polyethylene exchange versus full component revision for arthrofibrosis following total knee arthroplasty: A retrospective cohort analysis | Journal of Orthopaedics | United States | Retrospective comparative study | III | 2410 | To compare arthrolysis with polyethylene exchange vs full component revision for arthrofibrosis following primary TKA | Arthrofibrosis 100% | 2.0 (minimum) | Isolated polyethylene exchange with arthrolysis vs full component revision | Re-operation, Surgical complications | Adjusted models for re-operation within one- and two-years following isolated polyethylene exchange was significantly higher than full component revision (OR=1.52, CI 1.07–2.17, p=0.022 and OR=1.44, CI 1.06–1.97, p = 0.022) | Full component revision TKA for arthrofibrosis was associated with lower two-year re-operation rate than polyethylene exchange | Isolated polyethylene exchange |
| Cheng | 2024 | Clinical Outcomes of Isolated Polyethylene Exchange Versus Full Component Revision for the Management of Instability Following Total Knee Arthroplasty | Journal of Arthroplasty | United States | Retrospective comparative study | III | 280 | To compare the survivorship and patient-reported outcomes of patients undergoing revision TKA for instability with IPE versus full component revision | Instability 100% | 2.7 | Isolated polyethylene exchange vs full component revision | Re-revision, Implant survivorship, Radiographic assessment, Multiple PROMs | The survivorship for instability was significantly higher at 2 years (99 versus 92%, p=0.024) and 5 years (94 versus 84%, p=0.024) for patients undergoing full component revision | Isolated polyethylene exchange was not as reliable or durable compared to full component revision for the management of postoperative instability | Isolated polyethylene exchange |
| Chiu | 2009 | Antibiotic-impregnated cement in revision total knee arthroplasty. A prospective cohort study of one hundred and eighty-three knees | Journal of Bone & Joint Surgery | Taiwan | Randomised controlled trial | II | 183 | To evaluate the effect of vancomycin-impregnated cement on the prevention of deep infection after revision total knee arthroplasty | Aseptic loosening 73%, Component wear 27% | 7.4 | Standard vs antibiotic-impregnated cement | Superficial and deep infection rates, HSS score, Radiographic assessment | No deep infections in antibiotic-loaded cement group vs six (7%) in standard cement group (p=0.013) | Vancomycin-impregnated cement was shown to be effective in the prevention of postoperative deep infection after revision total knee arthroplasty | Cemented vs hybrid fixation |
| Cochrane | 2022 | Early Infection After Aseptic Revision Knee Arthroplasty: Prevalence and Predisposing Risk Factors | Journal of Arthroplasty | United States | Case-control study | III | 157 | To evaluate the prevalence of early PJI after aseptic revision TKA | Aseptic loosening 38%, Instability 21%, Arthrofibrosis 11%, Malalignment 10%, Polyethylene wear 9%, Other 11% | 3.8 | Aseptic rTKA. Risk factors for PJI evaluated incl. association with use of allograft, metaphyseal cones and bone cement | Prosthetic joint infection, differences in exposures | No association between use of allograft, metaphyseal cones or the number of components revised and PJI | Anaemia was an independent predictor of PJI, while technical factors did not have an association. | Zone 2 fixation devices |
| Crawford | 2017 | Results of a modular revision system in total knee arthroplasty | Journal of Arthroplasty | United States | Case series | IV | 278 | To evaluate midterm outcomes for revision TKA using a modular revision knee system with complete interchangeability and multiple options for augmentation, offset, constraint, and stem extensions | Aseptic loosening 47%, Instability 30%, Polyethylene wear 22%, Osteolysis 13%, Arthrofibrosis 12%, Malalignment 3%, Metallosis 3%, Fracture 2%, Other 7% | 6 | Aseptic rTKA using the Vanguard Super Stabilised Knee (SSK) modular revision system (Zimmer Biomet) | Re-revision, Implant survivorship, KSS, ROM, UCLA activity score, Radiographic assessment |  | The results of this modular TKA revision system at 6 years mean follow-up are promising for use in complex scenarios, with a low frequency of aseptic rerevision, good knee stability, and substantial improvements in range of motion and clinical and functional outcomes | Other- specific revision systems |
| Darbandi | 2025 | Short- and Mid-term Radiographic Outcomes of Ream-then-broaching Metaphyseal Cones During Revision Total Knee Arthroplasty | Arthroplasty Today | United States | Case series | IV | 144 | To assess the rates of short- and mid- term postoperative radiolucency for patients who underwent rTKA using a single metaphyseal cone design | Mechanical complications 61.1%, Instability 25.0%, Infection 9.7%, Progressive arthritis 2.1%, Dislocation 1.4%, Arthrofibrosis 0.7% | 2.2 | rTKA with placement of a metaphyseal cone utilising a Smith&Nephew ream-then-broach design | Re-revision, Radiographic assessment, Intraoperative fractures | No intraoperative complications were reported. No cases of cone or stem aseptic loosening were observed. | The utilisation of a ream-then-broach metaphyseal cones demonstrated excellent radiographic stability at short- and mid-term follow-ups. The use of this method has minimal risk of intraoperative or short-term failures. | Zone 2 fixation devices |
| Darwich | 2023 | Cemented versus Cementless Stem Fixation in Revision Total Knee Arthroplasty: A Systematic Review and Meta-Analysis | Antibiotics | Germany | Systematic review | II | 3203 | To compare cemented versus cementless stem fixation in rTKA regarding implant survivorship, failure rates and its causes, as well as clinical outcome of each of these fixation methods. | Not defined; heterogeneous | Mean range 1.5-10.2 | Cemented vs cementless stems | Aseptic loosening, Infection, Instability, Radiolucent lines | Rates of revision or failure for any reason as well as incidence of aseptic loosening and periprosthetic infection in cemented versus cementless stem fixation showed no significant difference. | No superiority of a specific stem fixation method in rTKA was found | Cemented vs hybrid fixation |
| Duensing | 2020 | Revisiting the role of isolated polyethylene exchange for aseptic failures in total knee arthroplasty | The Knee | United States | Case series | IV | 122 | To compare the difference in the cumulative incidence of reoperation after IPE five years after index surgery | Instability 52%, Polyethylene wear 34%, Arthrofibrosis 6%, Patella fracture 4%, Patella resurfacing 2%, Other 3% | 5.2 | Isolated polyethylene exchange for aseptic rTKA | Re-revision, Implant survivorship | 87% (95% CI, 78–92%) survivorship free of reoperation at five years. IPE for polyethylene wear was found to be protective and less likely to require reoperation (SHR 0.121 95% CI: 0.016–0.896, p = 0.039). | The current study suggests that when done for carefully selected indications, IPE may be an acceptable procedure and helpful alternative for aseptic TKA revisions, particularly when the pre-operative diagnosis is polyethylene wear. | Isolated polyethylene exchange |
| Eder-Halbedl | 2023 | Excellent mid- to long-term survival of tantalum metal cones in a case series of revision knee arthroplasty with severe bony defects | Knee Surgery, Sports Traumatology, Arthroscopy | Austria | Case series | IV | 100 | To evaluate the mid- to long-term survival for TMC and implants in a consecutive series of patients who underwent complex RTKA with severe bony defects | Aseptic loosening 45%, Infection 21%, Instability/Malrotation 17%, Polyethylene wear 14%, Fracture 3% | 6.1 | rTKA with TMC for tibia and/or femur bone defects | Re-revision, Implant survivorship, Re-operation, Complications, KSS, Radiographic assessment | At the final FU, 75 (94%) tibia and 76 (95%) femur TMCs and implants were clinically stable. There were eight (10%) reoperations including six (7.5%) re-revisions | Tantalum metal cones (TMC) demonstrate a secure fixation for treatment of severe femoral and tibial metaphyseal bone defects during RTKA. This fixation concept showed excellent mid- to long-term clinical and radiographic outcomes. | Zone 2 fixation devices |
| Engh | 2012 | Polyethylene quality affects revision knee liner exchange survivorship | Clinical Orthopaedics and Related Research | United States | Retrospective comparative study | III | 135 | To compare the re-revision rates and survivorship of isolated polyethylene exchange, partial revision, and full-revision procedures | Component wear/Osteolysis 100% | 6.2 | Isolated polyethylene exchange, partial revision, or full-revision procedures | Re-revision, Implant survivorship | Five-year survivorship was similar for all three procedures: 82% ± 14% for polyethylene exchange, 89% ± 8%for partial revision, and 88% ± 10% for a full revision. Polyethylene sterilization had the strongest influence on rerevision. Survivorship was 73% ± 16% for knees revised with gamma-in-air polyethylene compared to 92% ± 6% for nongamma or gamma-in-barrier sterilization methods | The survival rates of isolated polyethylene exchange for wear or osteolysis are similar to those of a single-component or full revision when the components are well aligned and well fixed. Polyethylene sterilization influenced revision TKA survivorship in this study and should be reported in future studies | Isolated polyethylene exchange; Single- vs full-component revision |
| Fawley | 2024 | Mid-term survivorship and clinical outcomes of revision knee arthroplasty using a mobile bearing tibial tray: A single-center registry review | The Knee | United States | Case series | IV | 341 | To review the survivorship and clinical outcomes from a company-sponsored joint replacement registry for a mobile bearing tibial tray used with metaphyseal sleeves | Instability 47%, Aseptic loosening 19%, Infection 14%, Pain/stiffness 4%, Fracture 3%, Dislocation/subluxation 1%, Other 12% | 7.0 (minimum) | Mobile Bearing Tibia (MBT) Revision knees (DePuy Synthes) | Re-revision, Implant survivorship | KM estimates (95% CI; n with further follow-up) for all-cause revision were 82.5% (75.8%; 87.5%; 42) at 7 years for the clinical assumption, and 88.5% (84.4%,91.6%; 53) at 13 years for the registry assumption | In this single-center registry evaluation, we found excellent mid-term survivorship and clinical outcomes for a mobile-bearing tibial tray used with metaphyseal sleeves | Zone 2 fixation devices; Other- Fixed- vs mobile- bearings |
| Fehring | 2005 | Outcome comparison of partial and full component revision TKA | Clinical Orthopaedics and Related Research | United States | Retrospective comparative study | III | 149 | To compare the clinical and radiographic results of partial-component versus full-component revision total knee arthroplasty | Aseptic loosening 34%, Instability 26%, Wear 22%, Stiffness 1%, Malalignment 1%, Fracture 3%, Miscellaneous 12% | 5.3 | Single- versus full-component revision | Re-revision, KSS, Radiographic loosening | There was no significant difference in rates of re-revision between the two groups (p=0.18). The average Knee Society score for full-component revisions was 85 compared with 79 for partial revisions (p=0.0001). The average Knee Society score for those patients who had a full revision for instability was 85 compared with 63 for partial revision (p = 0.0001) | Care should be taken when considering partial revision for instability or wear-related problems | Single- vs full-component revision |
| Fiedler | 2024 | Clinical Outcomes of Offset Stem Couplers with or without Cone Augmentation in Revision Total Knee Arthroplasty | The Journal of Knee Surgery | United States | Retrospective comparative study | III | 358 | To investigate clinical outcomes in rTKA using different fixation techniques | Not defined | 2.1 | Press-fit stem with an offset coupler (OS), fully cemented straight (CS) stem, and press-fit straight (PFS) stem | Re-revision, Re-admission, Surgical time, Length of stay | When stem type alone was considered, there was no significant difference in re-revision rate (p=0.431). Subanalysis demonstrated that augmentation with a tibial cone led to significantly higher rates of re-revision in the offset-stem group (OS=18.2% vs CS=2.1% vs PFS=11.1%; p=0.037) | Surgeons should consider using cemented stems in patients requiring tibial cone augmentation in rTKA. In patients not requiring tibial cone augmentation, surgeons may adhere to their preferred method of fixation without concern for inferior outcomes | Zone 2 fixation devices; Cemented vs hybrid fixation |
| Fleischman | 2017 | Effect of Stem Size and Fixation Method on Mechanical Failure After Revision Total Knee Arthroplasty | Journal of Arthroplasty | United States | Retrospective comparative study | III | 223 | To evaluate the risk for mechanical failure of revision TKA based on stem fixation method, stem size, and stem canal fill | Aseptic loosening 53%, Infection 26%, Instability 18%, Arthrofibrosis 3%, Component wear 1% | 5.1 | Fully cemented stems vs 'hybrid' press-fit stems | Re-revision, Infection, Radiographic assessment | Risk for mechanical failure was equivalent for both cemented and hybrid stems (relative risk, 0.991; p=0.98). Young age was the single greatest risk factor for mechanical failure (p=0.006). Although there was a trend toward increased failure with cemented stems in patients aged <65 years, there was no significant difference in risk after accounting for covariates (relative risk, 1.4; p=0.50). Intramedullary canal fill, not stem length or diameter, was the strongest predictor of failure with hybrid stems | Both cemented and hybrid modular stems are viable options in revision TKA. Surgeons should attempt to maximize canal filling of hybrid stems to obtain a solid press-fit. In addition, further studies are needed to evaluate the long-term survival of cemented stem fixation in young patients. | Cemented vs hybrid fixation |
| Frisch | 2026 | Midterm clinical outcomes and survivorship of a next generation revision knee system in complex primary and revision total knee arthroplasty | The Knee | United States | Prospective case series | IV | 185 | To evaluate the early clinical outcomes of a novel revision knee system for use in revision and complex primary TKA | Revision 90%, Complex primary 8%, Infection (Primary or revision) 3% | 2.5 | rTKA or complex primary TKA with condylar-constrained Persona® Revision Knee System implants and instrumentation | Implant survivorship, KSS, KOOS JR, Pain reduction | Implant survivorship at 2 years was 97.2% (95%CI 93.5–98.8) considering all indications; 97.0% (95%CI 93.0–98.7) in revision cases only. KS-KS scores improved significantly in all cohorts, with overall mean increase of 40.8 ± 22.9 points (p < 0.001). KOOS JR scores improved by 27.0±20.3 (p<0.001). Sub-group analysis of fixation method revealed no difference in KS-KS scores | This revision implant system demonstrated high survival and improvement in functional outcomes after 2 years, in both the complex primary and revision TKA setting | Constraint, Cemented vs hybrid fixation |
| Greenberg | 2024 | Prevalence and clinical impact of radiographic sclerotic lines adjacent to cementless tibial stems in revision total knee arthroplasty: a long-term follow-up study | European Journal of Orthopaedic Surgery & Traumatology | Canada | Case-control study | III | 153 | To quantify the prevalence of sclerotic lines at the bone-implant interface and assess the impact of progressive sclerotic lines on revision for aseptic loosening | Aseptic loosening 44%, Infection 16%, Malrotation 15%, Instability 12%, Fracture 8%, Stiffness 3%, Dislocation 1% | 16.5 | rTKA with the NexGen Legacy Constrained Condylar Knee implant system (LCCK, Zimmer Biomet), treated with a hybrid cementing technique at zone 2 and press-fit diaphyseal stems | Re-revision, Implant survivorship, Radiographic assessment | Survivorship free of failure due to aseptic loosening was 94.8% (95% CI 76.4–98) at 15 years, with three patients requiring re-revision surgery for aseptic loosening. There was no significant difference in rates of aseptic loosening in patients with or without sclerotic lines. | Radiographic sclerotic lines around revision TKA stems—measuring less than 2mm wide, nonprogressive, and located at the lateral-distal aspect of the stem—were identified in fewer than 8% of cases and did not correlate with re-revision surgery | Cemented vs hybrid fixation; Constraint |
| Greenberg | 2025 | Survivorship and Complications in Revision Total Knee Arthroplasty With a Constrained Condylar Knee Implant: A Minimum 10-Year Follow-Up Study | Journal of Arthroplasty | Canada | Case series | IV | 345 | To evaluate the survivorship and complications of CCK implants over a minimum 10-year follow-up, extending up to 23 years | Aseptic loosening 23%, Infection 23%, Malalignment 14%, Instability 13%, Pain/stiffness 6%, Fracture 5%, Other/Unknown 16% | 13.5 | rTKA with Legacy Constrained Condylar Knee (Zimmer Inc., Warsaw, Indiana) implants | Implant survivorship, Re-revision, Complications | All-cause revision-free survival was 95.1% (95% CI [confidence interval]: 92.7 to 97.5) at 5 years and 93.6% (95% CI: 91.0 to 96.2) from 10 years onward. The leading causes for re-revision were infection (54.5%) and aseptic loosening (31.8%). | The CCK implants demonstrate favorable long-term survivorship in rTKA at a minimum 10-year follow-up. These findings support the continued use of CCK implants in rTKA with appropriate patient selection and careful surgical technique | Constraint |
| Greene | 2013 | Midterm results of hybrid cement technique in revision total knee arthroplasty | Journal of Arthroplasty | United States | Case series | IV | 119 | To report results of revision TKA with uncemented intramedullary stems | Aseptic loosening 66%, Infection 24%, Instability 7%, Fracture 3%, Dislocation 2% | 5.2 | rTKA using Scorpio TS total knee system (Stryker) with Titanium smooth fluted stems (press-fit) | Re-revision, KSS, Radiographic assessment | Knee Society Pain and Function scores improved from 39 and 58 to 68 and 79 (p<0.05). Three knees (2.5%) required revision without any cases of aseptic loosening | At mid-term follow-up, revision hybrid TKA provides excellent fixation with extremely low rates of aseptic loosening and avoids the inherent complications of extensive intramedullary cement | Cemented vs hybrid fixation |
| Hajiaghajani | 2026 | Isolated Tibial or Femoral Component Revision Is Associated with Greater Revision Failure Rate Compared with Full Component Revision in Aseptic Revision Total Knee Arthroplasty: A Systematic Review and Meta-Analysis on Reconstructed Individual Participant Data | JBJS Reviews | Iran | Systematic review | II | 4429 | To compare full-component revision and isolated component revision in aseptic rTKA regarding all-cause and cause-specific revision failures, postoperative range of motion (ROM), and patient-reported outcome measures (PROMs) | Not clearly defined; heterogeneous | Range 2.0-10.0 | Single- versus full-component rTKA | Re-revision, ROM, PROMs | Single-component revision was associated with a higher hazard of all-cause re-revision (OR 1.58; 95% CI 1.21-2.07; p < 0.001). Cause-specific analyses were imprecise and crossed the null for aseptic loosening (OR 1.50; 95% CI 0.75-2.99), infection related failure (OR 0.90; 95% CI 0.41-1.96), and instability-related failure (OR 1.03; 95% CI 0.43-2.46). PROMs did not clearly differ between strategies | While single-component revision is associated with a higher all-cause revision failure rate, the choice between techniques remains subject to surgeon discretion and patient-specific factors | Single- vs full-component revision |
| Hanusrichter | 2026 | Fast fix or false hope? Polyethylene exchange in unstable primary unconstrained total knee arthroplasty | Bone & Joint Open | Germany | Case series | IV | 1890 | To assess outcomes and risk factors of isolated polyethylene exchange for secondary instability in unconstrained primary TKAs using data from the German Arthroplasty Registry (EPRD). | Instability 100% | 2.8 | Isolated polyethylene exchange in unstable primary unconstrained total knee arthroplasty | Re-revision | The overall re-revision rate was 13.5%; 2.4% were due to periprosthetic joint infection. Secondary patella resurfacing was associated with lower revision risk (hazard ratio (HR) 0.50; 95% CI 0.37 to 0.68; p < 0.001). Polyethylene height increase >3 mm was not associated with worse outcomes (< 3 mm HR 0.87, 95% CI 0.65 to 1.17; p=0.358) | IPE can be a viable treatment for mild secondary instability following primary TKA, particularly with careful patient selection. SPR appears to improve implant survival and should be considered in cases with patellar pathology | Isolated polyethylene exchange |
| Hardeman | 2012 | Predisposing factors which are relevant for the clinical outcome after revision total knee arthroplasty | Knee Surgery, Sports Traumatology, Arthroscopy | Belgium | Case series | IV | 146 | To evaluate the effect of the reason for index failure, as well as a number of well-defined characteristics of the index procedure, on the actual outcome after rTKA | Aseptic loosening 25%, Instability 23%, Infection 14%, Malalignment 11%, Component wear 10%, Patella complications 6%, Arthrofibrosis 4%, Fracture 3%, Other 4% | 4.8 | rTKA. Re-revision risk factors evaluated incl. single- vs full-component revision | Implant survival, KSS, Radiographic assessment | The cause of index failure had no significant influence on any of the outcome parameters. Significantly, better outcomes were noted for partial revisions and for revisions in older patients. | Revision TKA leads to a significant reduction in symptoms and improvement in function. The worst results can be expected for early revisions in young patients. | Single- vs full- component revision |
| Heesterbeek* | 2016 | No Difference in Implant Micromotion Between Hybrid Fixation and Fully Cemented Revision Total Knee Arthroplasty: A Randomized Controlled Trial with Radiostereometric Analysis of Patients with Mild-to-Moderate Bone Loss | Journal of Bone & Joint Surgery | Netherlands | Randomised controlled trial | II | 32 | To compare cemented and hybrid constructs in terms of implant stability and clinical outcome | Component malposition/malrotation 31%, Instability 25%, Aseptic loosening 22%, Patella subluxation 9%, Infection 9%, Polyethylene wear 3% | 2 | Cemented vs press-fit stems | KSS, KOOS, VAS scores, Radiostereometric analysis, Adverse events | At 24 months, no difference in median migration or the number of migrating components was found. The clinical scores did not differ between techniques. | Cemented and hybrid-fixation replacements were equally stable. Both groups had implants with >1mm or >1deg of micromotion; whether this indicates the possibility of loosening with longer follow up remains to be investigated. | Cemented vs hybrid fixation |
| Hinman | 2025 | Impact of Metaphyseal Sleeves and Porous Cones on Risk Reduction for Revision Total Knee Arthroplasty: Aseptic Re-Revision in a United States Integrated Health Care System | Journal of Arthroplasty | United States | Retrospective comparative study | III | 2882 | To compare rates of aseptic all-cause re-revision and aseptic re-revision specifically for loosening of rTKA performed with modular stems alone versus those with both modular stems and metaphyseal cones or sleeves | Infection excluded, otherwise not clearly defined | 5.8 | rTKA with stems alone vs rTKA with stems and cones/sleeves | Re-revision | At 8-year follow-up, after propensity score weighting, no difference was observed between stem + cone/sleeve compared with stem alone for aseptic re-revision (hazard ratio=0.76, 95% confidence interval=0.42 to 1.37) or aseptic loosening specifically (hazard ratio=0.62, 95% confidence interval=0.27 to 1.43) | The addition of metaphyseal sleeves or porous cones did not change the risk of aseptic re-revision overall or re-revision due to loosening specifically. Further large-scale studies with long-term follow-up are warranted to confirm these results. | Zone 2 fixation devices |
| Hoskins | 2026 | The AAHKS Surgical Techniques and Technologies Award: What Is the Ideal Technique and Implant in Aseptic Revision Total Knee Arthroplasty? An Analysis of Second Revision and Component Loosening Rates From the Australian Orthopaedic Association National Joint Replacement Registry | Journal of Arthroplasty | Australia | Case series | III | 22238 | To assess the cumulative percent second-revision (CP2R) rates based on what components were revised and the stem extensions and porous metal augments (cones/sleeves) used | Aseptic indications 100%; Infection excluded | Mean range 4.0-5.2 | First-time aseptic rTKA with one of 14 revision constructs; full-component revision with tibial and femoral stems and either cones or sleeves was used as the reference group | Re-revision | When compared with the referent, complete revision with no stems or cones/sleeves had a statistically higher risk of all-cause revision (HR 2.42, 95% CI: 1.41 to 3.98, P < 0.001). However, statistical evidence was lacking for the other three complete revision constructs (HR 1.19 to 1.28; p=0.13 to 0.36). In terms of revision for loosening, all three complete revision groups with adequate numbers had a higher risk of being revised (P-value range < 0.001 to 0.07) | Complete component revision using both stems and cones or sleeves has the lowest re-revision rates for all causes and specifically for component loosening. Enhanced fixation with stems and cones or sleeves should be considered strongly for rTKA. | Single- vs full-component revision; Zone 2 fixation devices; Isolated polyethylene exchange |
| Howard | 2023 | Comparison of Clinical and Functional Outcomes in One versus Two Component Revision for Total Knee Arthroplasty | Journal of Arthroplasty | Canada | Retrospective comparative study | III | 262 | To compare 1- versus 2-component revisions for TKA with a focus on complications, rerevisions, survivorships, and functional outcomes | Aseptic loosening 62%, Instability 16%, Stiffness 10%, Malalignment 3%, Polyethylene wear 3%, Other 6% | 8.7 | Single vs both component rTKA | Re-revision, Surgical complications, Operative times, Multiple PROMs | The overall complication prevalence in the 1- and 2- component revision groups was similar 20 of 92 (22%) and 35 of 170 (21%), respectively (p=0.87). There was no statistical difference in survivorship or functional outcomes scores between the groups | Isolated revision of a single TKA component is an acceptable option, with comparable functional outcomes, complications, and survivorships when compared with both component revision | Single- vs full-component revision |
| Hurtado-Oliver | 2024 | Etiology and prosthesis model have no influence in the functional outcomes of total knee arthroplasty revision surgery at five years | Journal of Clinical Orthopaedics and Trauma | Spain | Retrospective comparative study | III | 108 | To compare the functional outcomes of patients undergoing total knee replacement revision surgery in terms of KSS score, pain, knee range of motion, and independence for walking according to the cause of the revision, septic or aseptic, and the prosthesis model implanted at 5 years | Aseptic 71.3%, Septic 28.7% | 5 | Three different models of revision prostheses; Vanguard® 360 Revision Knee System (Biomet® Orthopedics, Warsaw, Indiana, USA), Triathlon® TS Knee System (Stryker® Orthopedics, Mahwah, NJ, USA), and Dual Articular Knee AGC® Total Knee System (Biomet® Merck Limited, UK) | KSS, ROM, Walking status, Pain | 81.5% demonstrated good or excellent functional outcomes with a mean KSS score of 76.7 ± 14,3, a mean ROM of 1.68 ± 0.30 radians, and 77% independent for walking without any aids. There were no differences between the three revision systems. | TKA revision surgery offers good clinical and functional outcomes, independence for walking, good range of motion, and low residual pain regardless of the cause of the revision or the model of prosthesis. | Other- specific revision systems |
| Jaibaji | 2022 | Metaphyseal fixation in revision knee arthroplasty: a systematic review of the literature and meta-analysis of mid-long-term outcomes of metaphyseal sleeves and cones | Acta Orthopaedica Belgica | United Kingdom | Systematic review | II | 1431 | To analyse the mid to long term results of porous metaphyseal cones and sleeves as well as compare their outcomes. | Not defined; heterogeneous | 6.2 | rTKA with metaphyseal cones or sleeves | Re-revision, Re-operation | Metaphyseal cones demonstrated a higher re-revision rate when compared to sleeves 10.85% vs 6.31 (p=0.007). Reoperation rates were also higher in cones compared to sleeves, 13.78% vs 3.68% (p<0.001). Prosthetic joint infection was the most common reason for re-revision. | Metaphyseal sleeves demonstrate significantly lower re-revision rates compared to metaphyseal cones. However, overall, both demonstrate reliable mid- to long-term outcomes | Zone 2 fixation devices |
| Jensen | 2012 | Bone mineral density changes of the proximal tibia after revision total knee arthroplasty. A randomised study with the use of porous tantalum metaphyseal cones | International Orthopaedics | Denmark | Randomised controlled trial | II | 40 | To measure quantitatively using DEXA the adaptive bone remodelling of the proximal tibia and tibial shaft after rTKA with or without a TM cone | Aseptic loosening 39%, Infection 28%, Instability 17%, Pain 11%, Polyethylene wear 6% | 2 | rTKA with or without a TM cone | Bone mineral density, KSS | Overall decreases in BMD of 0.1-5.4% were found in both groups at 12 months. By 24 months, an increase in BMD was found along the stem of 1.9-6.3%, with significant changes in the TM cone group. There were no significant differences in BMD changes of KSS scores between the groups. | The bone remodelling pattern was almost the same in the two groups after two years | Zone 2 fixation devices |
| Kemker | 2022 | Comparing Revision Total Knee Arthroplasty Stems at a High-Volume Revision Center | Frontiers in Surgery | United States | Retrospective comparative study | III | 133 | To compare cemented and hybrid stems in rTKA, looking primarily at survivorship at final follow-up | Not defined; heterogeneous | 2.1 | Cemented vs hybrid fixation | Implant survivorship, Length of stay, Change in Hb | Overall survival rates were 80.9% in the hybrid and 84.6% in the cemented groups (p = 0.642). Mean LOS (hybrid 4.13 days, cemented 3.65 days; p = 0.356) and change in Hgb(hybrid 2.95 mg/dL, cemented 2.62mg/dL; p = 0.181) were not statistically different between the groups | Hybrid and fully cemented rTKA techniques have similar survival rates at a minimum followup of 2 years. Continued long-term research is required for defining the best rTKA technique. | Cemented vs hybrid fixation |
| Kim | 2018 | Comparative Analysis between Cone and Sleeve in Managing Severe Bone Defect during Revision Total Knee Arthroplasty: A Systematic Review and Meta-Analysis | The Journal of Knee Surgery | South Korea | Systematic review | II | 775 | To compare the outcomes between revision TKA performed using a tantalum cone and that using titanium sleeve | Not defined; heterogeneous | 2.6 | Tantalum cones vs titanium sleeves in rTKA | Multiple PROMs, Surgical complications, Radiographic assessments | The knee scores of the cone and sleeve groups were increased in all studies with varying statistical significance. Radiolucent lines were seen in six cases in the cone group and in 15 cases in the sleeve group. Only a few specific augment-related complications were noted. | Results support the use of the newly devised augments in bone defect during revision TKA in terms of clinical and radiological outcome | Zone 2 fixation devices |
| Kim | 2017 | Midterm Clinical and Radiographic Results of Mobile-Bearing Revision Total Knee Arthroplasty | Journal of Arthroplasty | United States | Case series | IV | 316 | To (1) evaluate the Knee Society (KS) scores and range of motion in patients undergoing revision TKA using mobilebearing revision knee implants, (2) assess the midterm radiographic results, and (3) evaluate the survivorship, complications, revisions, and reoperations, including the incidence of bearing complications | Aseptic loosening 30%, Instability 29%, Infection 16%, Faile unicompartmental replacement 8%, Arthrofibrosis 8%, Polyethylene wear 4%, Other 5% | 4.9 | rTKA with mobile-bearings | Re-revision, KSS, Radiographic assessment, Surgical complications | The average Knee Society knee score and function scores increased from 40.8 and 47.9 points preoperatively to 80 points and 70.3 points, respectively (p<0.01). The average knee flexion improved from 105.6 preoperatively to 117.4 postoperatively (p<0..01). Eight patients required subsequent implant revision. No cases of bearing complications were observed. | Revision TKA using mobile-bearing revision components demonstrated favorable midterm clinical and radiographic results with no occurrence of bearing instability or dislocation. Longer followup is required to evaluate for potential advantages of mobile-bearings over fixed-bearing revision components | Other- Fixed- vs mobile- bearings |
| Kim | 2020 | Long-Term (Up to 21 Years) Survival of Revision Total Knee Arthroplasty with Use of a Constrained Condylar Knee Prosthesis: A Concise Follow-up of a Previous Report | Journal of Bone & Joint Surgery | South Korea | Case series | IV | 114 | To determine the long-term clinical and radiographic results of revision with use of the LCCK prosthesis (Zimmer Biomet) | Aseptic loosening 54%, Polyethylene wear 22%, Infection 12%, Instability 8%, Other 4% | 19.2 | rTKA with Legacy constrained condylar prosthesis (LCCK; Zimmer Biomet) | Re-revision, Implant survivorship, Radiographic assessment, Multiple PROMs | Re-revision had been performed in 10 knees(9%). The rate of survival at 19.2 years was 96% (95% confidence interval [CI], 94% to 100%) with reoperation for mechanical failure as the end point, and 91% (95% CI, 87% to 98%) with reoperation for any reason as the end point. | rTKA with the LCCK provided good functionality, patient satisfaction, and implant survival in 91% of patients at a mean follow-up of 19.2 years | Constraint |
| Kim | 2009 | Revision total knee arthroplasty with use of a constrained condylar knee prosthesis | Journal of Bone & Joint Surgery | South Korea | Case series | IV | 114 | To ascertain the role of the constrained condylar knee prosthesis and to determine whether it can provide satisfactory results after rTKA | Aseptic loosening 54%, Polyethylene wear 22%, Infection 12%, Instability 8%, Other 4% | 7.2 | rTKA with Legacy constrained condylar prosthesis (LCCK; Zimmer Biomet) | Re-revision, Implant survivorship, Multiple PROMs, Surgical complications | All PROMs improved postoperatively. The complication rate was 9%. Five knees (4%) had a re-revision. 10-year KM survivorship was 96% (CI=94%-100%) | Revision total knee arthroplasty with use of a constrained condylar knee prosthesis had reproducible clinical success, but a complication rate of up to 9% can be expected at intermediate-term follow-up | Constraint |
| Klasan | 2020 | Factors predicting repeat revision and outcome after aseptic revision total knee arthroplasty: results from the New Zealand Joint Registry | Knee Surgery Sports Traumatology Arthroscopy | New Zealand | Case series | IV | 1720 | To analyse factors predicting repeat revision following aseptic revision TKA | Pain/Other 36%, Aseptic loosening 34%, Instability 12%, Stiffness 7%, Malalignment 4%, Poly failure 4%, PFJ arthritis 3%, AVN 1%, Extensor mechanism deficiency 1% | Not stated | Isolated tibial-, femoral-, or full-component revision | Re-revision, OKS | Femoral component revision was a predictor of re-revision (HR 1.696) and had the lowest OKS, compared to tibial and all component revision (p=0.003) | Isolated femoral component exchange led to worse outcomes both in terms of survivorship and functional scores | Single- vs full-component revision |
| Kosse* | 2017 | Comparable Stability of Cemented vs Press-Fit Placed Stems in Revision Total Knee Arthroplasty With Mild to Moderate Bone Loss: 6.5-Year Results From a Randomized Controlled Trial With Radiostereometric Analysis | Journal of Arthroplasty | Netherlands | Randomised controlled trial | II | 23 | To examine the midterm results of cemented vs hybrid-placed stems in patients after revision TKA, in terms of implant stability and clinical outcomes. | Not clearly defined; heterogeneous | 6.5 | Cemented vs press-fit stems | KSS, KOOS, VAS scores, Radiostereometric analysis, Adverse events | There were no differences in median total translation and rotation of the femoral and tibial components between the 2 groups, and none of the clinical scores differed between the groups. Five cemented tibia implants showed >1 mm/degree micromotion compared to none in the hybrid group | After 6.5-year follow-up, micromotion of the fully cemented constructs is comparable to that of the hybrid-placed constructs. Two femur components showed progressive migration over time whereas tibia components migrate especially in the first year after implantation followed by a stable situation. Whether this will lead to loosening and re-revisions remains to be investigated | Cemented vs hybrid fixation |
| Kunze | 2019 | Superior Survivorship for Posterior Stabilized Versus Constrained Condylar Articulations After Revision Total Knee Arthroplasty: A Retrospective, Comparative Analysis at Short-Term Follow-Up | Journal of Arthroplasty | United States | Retrospective comparative study | III | 253 | To investigate and compare implant survival, complications, and radiologic and clinical outcomes of patients who underwent rTKA with a CCK articulation design to those with a PS articulation design at a minimum of 2-year follow-up | Aseptic loosening 59%, Infection 13%, Instability 11%, Acquired deformity 11%, Arthrofibrosis 4%, Trauma 3% | 3.5 | Posterior stabilised vs condylar constrained rTKA | Re-revision, Implant survivorship, KSS, ROM, Medical and surgical complications, Radiographic assessment | PS articulation was an independent predictor of increased postoperative knee flexion (6.4deg, p=0.010) and the knee society functional score (10.0, p=0.002). Survivorship was significantly reduced for CCK revision articulations when all-cause re-revision was the primary endpoint (p=0.0003). PS articulations conferred a lower likelihood of re-revision (OR=0.3, p=0.001), but articulation design was not associated with complications (OR=0.5, p=0.123) or aseptic loosening (OR=2.6, p=0.143) | The PS articulation when used for appropriate indication conferred superior survivorship for the primary endpoint of all-cause re-revision and overall knee function when compared to the CCK articulation after RTKA. Implant articulation was not a predictor of aseptic loosening or complications | Constraint |
| Larson | 2021 | Pain associated with cemented and uncemented long-stemmed tibial components in revision total knee arthroplasty | The Bone & Joint Journal | United States | Retrospective comparative study | III | 110 | To evaluate overall pain levels, diaphyseal tibial pain, and patient satisfaction in revision TKA by comparing matched cohorts receiving long- stemmed uncemented or cemented tibial components | Aseptic loosening 35%, Infection 26%, Instability 17%, Arthrofibrosis 11%, Component malrotation 9%, Other 4% | 2.0 (minimum) | rTKA with cemented vs uncemented stemmed tibial components | Postal survey incl. patient satisfaction score and modified KSS (pain/tibial pain), | Tibial pain at the end of the stem was present in 25.3% (16/63) of cemented stems and 25.5% (12/47) of uncemented stems (p = 1.000); 74.6% (47/63) of cemented patients and 78.7% (37/47) of uncemented patients were satisfied following revision TKA (p = 0.657) | There were no differences in patient satisfaction, overall pain, and diaphyseal tibial pain in cemented and uncemented stemmed tibial components in revision TKA. Patient factors, rather than implant selection and surgical technique, likely play a large role in the presence of postoperative pain. | Cemented vs hybrid fixation |
| Lazic | 2022 | Modular Augmentation in Varus-Valgus-Constrained Knee Arthroplasty-Do We Need Sleeves to Avoid Femoral Loosening After Excessive Distal Augmentation? | Journal of Arthroplasty | Germany | Retrospective comparative study | III | 136 | To retrospectively investigate implant survival in relation to distal femoral augment heights and the presence of metaphyseal sleeve implantation | Instability 35%, Aseptic loosening 25%, Infection 24%, Polyethylene wear 6%, Periprosthetic fracture 1% | 4.2 | 4, 8, and 12mm distal femoral augments; subgroup analysis for femoral sleeve implantation | Re-revision, Implant survival | Implant survival of 97.0%, 87.5%, and 69.2% for 4, 8, and 12mm distal femoral augmentation, respectively (p=0.73; p=0.19; p=0.008). Implant survival with femoral sleeves was 95.8% for the 8 mm augments and 85.7% for the 12 mm augments (p=0.42; p=.96). Without femoral sleeves, implant survival was 78.3% with the 8mm augments and 50.0% with the 12mm augments (p=0.02; p=0.001) | In cases with femoral metaphyseal bone damage requiring high distal femoral augmentation, metaphyseal sleeves should be used to avoid early aseptic femoral loosening. | Zone 2 fixation devices |
| Leal | 2025 | Comparing Survivorship of Symmetric Versus Asymmetric Tibial Augments in Aseptic Revision Total Knee Arthroplasty | Journal of Arthroplasty | United States | Retrospective comparative study | III | 103 | To evaluate radiolucency in patients who underwent aseptic revision total knee arthroplasty using either symmetric or asymmetric modular tibial augments and compare 5-year all-cause and nonseptic revision-free survivorship between groups | Pain 42%, Aseptic loosening 26%, Multifactorial 10%, Implant failure 8%, Instability 8%, Fracture 3%, Wear 3%, Other 21% | 4 | Symmetric versus asymmetric tibial augments at aseptic rTKA | Implant survivorship, Radiolucency | The 5-year all-cause and nonseptic revision-free survival were lower in patients who had asymmetric tibial augments compared to symmetric (56.1 versus 85.5%; p=0.021 and 64.8 versus 89%; p=0.019, respectively). Asymmetric tibial augment constructs with metaphyseal cones had similar 5-year nonseptic revision-free survivorship to symmetric tibial augment constructs with (75.0 versus 97.0%; p=0.13) or without metaphyseal cones (75.0 versus 82.5%; p=0.54) | Patients who had symmetric tibial augment constructs exhibited similar rates of radiolucency but higher survivorship compared to those with asymmetric constructs overall. However, when metaphyseal cones were utilized, survivorship was equivalent | Other- augments, Zone 2 fixation devices |
| Leta | 2015 | Failure of aseptic revision total knee arthroplasties | Acta Orthopaedica | Norway | Retrospective comparative study | III | 1016 | To analyse the survival rate of revision TKAs and to study the causes of failure of revision TKAs based on data in a national registry | Aseptic loosening 55%, Instability 24%, Malalignment 13%, Polyethylene wear 12%, Pain 8%, Arthrofibrosis 6%, Fracture 6%, Other 12% | 4.5 | Aseptic rTKA. Risk factors for re-revision evaluated incl. implant characteristics | Re-revision, Implant survivorship | The cumulative survival rate for revision total knee arthroplasties was 85% at 5 years, 78% at 10 years, and 71% at 15 years. Revision total knee arthroplasties with exchange of the femoral or tibial component exclusively had a higher risk of re-revision (RR = 1.7) than those with exchange of the whole prosthesis. There were no significant differences in survival rate between fixation type, constraint level or stem use. | In terms of implant survival, revision of the whole implant was better than revision of 1 component only. Young age and male sex were risk factors for re-revision. | Single- vs full-component revision; Cemented vs hybrid fixation; Constraint |
| Lewis | 2022 | Primary Total Knee Arthroplasty Revised for Instability: A Detailed Registry Analysis | Journal of Arthroplasty | Australia | Retrospective comparative study | III | 2605 | To: (1) document the frequency of TKA revised for instability, and the patient and prosthesis factors associated with this revision diagnosis; (2) describe the procedures used to treat TKA instability; and (3) compare the outcome of these surgical strategies by calculating the cumulative rate of re-revision | Instability 100% | Not stated | rTKA for instability. Risk factors for re-revision evaluated incl. implant characteristics | Re-revision | After a revision for instability, 24% had a second revision by 14 years. Recurrent instability accounted for 32% of further revisions. A lower second revision rate was seen after revision of both femoral and tibial components, and where constrained components were used | Revision for instability is increasing. Revising both femoral and tibial components led to a lower rate of second revision compared to a change in insert alone. Recurrent instability was common. | Isolated polyethylene exchange; Constraint |
| Limberg | 2020 | Varus-valgus constraint in 416 revision total knee arthroplasties with cemented stems provides a reliable reconstruction with a low subsequent revision rate at early to mid-term review | The Bone & Joint Journal | United States | Case series | IV | 416 | To determine the mid- term survivorship, complications, and clinical outcomes of a single, fixed- bearing VVC device in a large cohort of revision TKAs. | Instability 29%, Aseptic loosening 25%, Infection 23%, Other 23% | 4 | rTKA with a single varus-valgus constrained implant (Triathlon TS; Stryker) | Re-revision, KSS, ROM | The five- year cumulative incidence of any subsequent revision was 14% (95% CI 10 to 18, number at risk = 150). The use of this implant without stems was found to be a significant risk factor for subsequent revision (hazard ratio (HR) 7.58 (95% CI 3.98 to 16.03); p = 0.007). No relationship was identified between the use of cones and subsequent re-revision. | The cumulative incidence of subsequent revision for aseptic loosening and instability was very low at five years with this fixed- bearing VVC implant in revision TKAs. Routine use of cemented and stemmed components with targeted use of metaphyseal cones likely contributed to this low rate of aseptic loosening. | Constraint; Cemented vs hybrid fixation; Zone 2 fixation devices |
| Lin | 2024 | Isolated aseptic loosening in total knee arthroplasty: a comprehensive 10-year review of partial vs. total component revisions | BMC Musculoskeletal Disorders | Taiwan | Retrospective comparative study | III | 112 | To elucidate the efficacy of partial component revision verus total component revision in rTKA for isolated aseptic loosening | Aseptic loosening 100% | 6.8 | Partial component revision vs total component revision | Re-revision, Implant survivorship, Operative time, Blood loss, Length of stay | Partial component revision was associated with shorter operative times and hospital stays compared to total component revision, however, no significant differences in overall survivorship were observed between the groups | Both partial- and total- component revision provide similar survival rates and clinical outcomes for managing isolated aseptic loosening in TKA. Partial component revision provides advantages in terms of surgical efficiency and patient recovery, while reducing the need for more constrained prosthetic solutions. | Single- vs full-component revision |
| Lindberg-Larsen | 2014 | Re-admissions, re-operations and length of stay in hospital after aseptic revision knee replacement in Denmark | Bone & Joint Journal | Denmark | Retrospective comparative study | III | 1218 | To determine differences in early morbidity after aseptic revision knee replacement in relation to the extent of the procedure (total revisions, large partial revisions, partial revisions and revision of unicompartmental knee replacement (UKR revisions)) and to investigate potential risk factors associated with re-admissions, re-operations and LOS | Aseptic loosening 42%, Instability 25%, Pain without loosening 22%, Polyethylene failure 8%, Secondary patella resurfacing 7%, Other 15% | 0.3 | Total revisions (femoral and tibial components revised), large partial revisions (femoral or tibial component revised), partial revisions (femoral and tibial components retained) and revisions of unicondylar replacements | Re-admissions, re-operations and length of stay | The age ranges of 76 to 80 years (p=0.018) and the large partial revision subgroup (p=0.073) were related to an increased risk of re-operation | A tendency towards increased early morbidity after large partial revisions with a higher 90-day re-operation rate (p = 0.072) calls for further investigation focusing on the surgical technique and indications for the large partial procedure | Single- vs full-component revision |
| Lonner | 2003 | Fate of the unrevised all-polyethylene patellar component in revision total knee arthroplasty | Journal of Bone & Joint Surgery | United States | Case series | IV | 202 | To determine the intermediate-term fate of well-fixed and well-aligned all-polyethylene patellar components that were retained during revision tibiofemoral arthroplasty | Infection excluded, otherwise not clearly defined | 7 | rTKA in which a well-fixed, well-positioned all-polyethylene patellar component with minimal wear was retained | Re-operation, KSS, Anterior knee pain, Patellofemoral complications | KSS improved postoperatively. 68% of the revision tibiofemoral components were from a different manufacturer than the retained patellar component. 21 patients (10%) reported anterior knee pain and 17/21 underwent further surgery to address the source of the pain. Patella component failure was significantly greater when the component had been gamma irradiated in air than when sterilised with another method (p=0.0008). | Retaining a well-positioned, stable all-polyethylene patellar component at the time of revision tibiofemoral arthroplasty can be successful, provided that the polyethylene has not oxidized. Manufacturing mismatch is acceptable with most contemporary designs provided that the patellar component articulates appropriately with the femoral implant. | Other- management of patella at tibiofemoral rTKA |
| Lopez Lopez | 2024 | [Translated article] A systematic review of the literature: The use of metaphyseal sleeves in revision total knee arthroplasty | Revista espanola de cirugia ortopedica y traumatologia | Spain | Systematic review | II | 1666 | To analyse the indications, complications, survival of the implants and the clinical and functional result of the prosthetic revision surgery where metaphyseal sleeves were used | Aseptic loosening 44%, Infection 27%, Instability 16%, Other 13% | 4.9 | rTKA with the use of metaphyseal sleeves | Re-operation, Implant survivorship, Multiple PROMs | The included studies showed good both functional and clinical outcomes. The overall reoperation rate was 16.2%, with an overall survival rate of 92.2% and aseptic survivorship of 98.2% | Metaphyseal sleeves are a good treatment option for this surgery, especially in AORI II or III type bone defects, achieving good intraoperative and primary stability of the implant, with good and rapid osseointegration. | Zone 2 fixation devices |
| Luque | 2014 | Predictive factors for failure after total knee replacement revision | International Orthopaedics | Spain | Case series | IV | 125 | To assess the survivorship, the complications, the clinical, radiological, and functional situation, and the quality of life of those patients in whom an Optetrak Condylar Constrained revision total knee arthroplasty had been implanted | Aseptic loosening 100% | 9 | rTKA with Optetrak Condylar Constrained revision total knee arthroplasty | Implant survival, KSS, OKS, Surgical complications, Radiographic assessment | The mean results of the KSS clinical and the KSS functional were 68.24 and 63.85, respectively. There were not any conditions associated with poor results of the knees (p>0.05). The global survival at 24 months was 92.7 %, at 60 months 87.8% and at 96 months it was 87.8%. | Based on these results there are some preoperative factors that change the survival of the total knee replacement revision | Constraint |
| Lygrisse | 2021 | Factors Affecting Range of Motion After Revision Total Knee Arthroplasty | Journal of Arthroplasty | United States | Retrospective comparative study | III | 290 | To determine the effect that the type of revision, implant constraint level, and the reason for revision have on postoperative ROM after rTKA | Aseptic loosening 38%, Instability 27%, Arthrofibrosis/stiffness 20%, Implant failure 5%, Liner wear 5%, Other 5% | 1.0 (minimum) | Aseptic rTKA | Pre-operative, 3 month and 1 year postoperative ROM | ROM increased more in association with full component revision vs liner exchange alone (p=0.03). Change in ROM was not significant when comparing the level of implant constraint nor when separating and comparing by type of revision. | Component revision cases have significantly improved ROM when compared with liner-only revision. Constraint level is not significantly associated with changes in ROM in either liner or component revisions. Component revisions due to instability significantly reduce improvements in ROM. | Isolated polyethylene exchange; Constraint |
| Madanipour | 2025 | Outcomes of Liner Exchange Versus Component Revision for the Treatment of Stiffness Following Primary Total Knee Arthroplasty | Journal of Arthroplasty | Canada | Retrospective comparative study | III | 129 | To compare outcomes of rTKA with liner exchange versus component revision for stiffness post-TKA | Stiffness 100% | 8.5 | Isolated polyethylene exchange vs full component revision | Implant survivorship, ROM, Operative time, Blood loss, Length of stay | The mean gain in arc of motion was similar between liner exchange and component revision cohorts (p=0.45). The 10-year rerevision-free survival was similar between the liner exchange (86.2%) and component revision (81.1%) cohorts (p=0.55). Survival from re-revision for stiffness at 10 years was also similar (p=0.6) | Liner exchange resulted in similar achievement of a successful ROM and re-revision-free survival compared with component revision. In properly selected patients, liner exchange is a reasonable option for the treatment of stiffness after TKA. | Isolated polyethylene exchange |
| Maestri | 2023 | Isolated Femoral or Tibial Component Revision in Total Knee Arthroplasty: A Systematic Review | Medical Principles and Practice | Italy | Systematic review | II | 600 | To define when partial component revision is better than total component revision | Aseptic indications 100%; infection excluded | 4.5 | Partial component revision vs total component revision | Re-revision, Multiple PROMs, Radiographic assessment | Total revisions performed for instability and wear achieved better clinical outcomes: in the other cases, partial and total revisions showed no differences in clinical outcomes. The re-revision rates were similar in most of comparative studies: only one study noticed a significant difference in the failure rate between partial (25% at 3 years) and full (7% at 3.5 years) revisions. | The poor quality of the studies precluded sound conclusions. Isolated tibial or femoral component revision is an option when the other component is well fixed and positioned and in absence of chronic periprosthetic infection. | Single- vs full- component revision |
| Malviya | 2012 | Functional outcome following aseptic single-stage revision knee arthroplasty | Knee Surgery, Sports Traumatology, Arthroscopy | United Kingdom | Prospective comparative study (non-randomised) | III | 175 | To compare the patientreported outcome following aseptic revision knee replacement surgery between two different levels of constraint (non-linked constrained and linked constrained designs) | Aseptic loosening 46%, Instability, 27%, Polyethylene wear 13%, Unexplained pain 9%, Other 5% | 4 | rTKA with non-linked constrained vs linked constrained designs | WOMAC, SF 36v2, Postoperative satisfaction | All patients were found to have significant improvements in PROMs. WOMAC scores were significantly worse for patients revised for instability (27%) than for aseptic loosening (46%). Revision to a higher level of constraint did not improve knee function irresepective of the primary indication for surgery. | This study has found that revision for instability, irrespective of choice of new device, was met with significantly poorer functional outcome. The level of constraint did not influence functional outcome. | Constraint |
| Martin | 2020 | Is a full component revision necessary for isolated tibial loosening? | The Bone & Joint Journal | United States | Retrospective comparative study | III | 164 | To compare the outcome of isolated tibial revision with that of full component revision for aseptic tibial loosening | Aseptic loosening 100% | 3.5 | Isolated tibial component revision vs full component revision | Re-revision, Re-operation, Surgical complications, Radiographic assessment | Supplementary femoral metaphyseal fixation was required in five patients in the full revision cohort. There was a higher incidence of radiological tibial loosening in the full component revision cohort at the final follow- up (8 (10.5%) vs 5 (5.7%); p = 0.269). Three patients in the full component revision cohort developed instability while only one in the isolated tibial cohort did. Three patients in the full revision cohort developed a flexion contracture greater than 5˚ while none in the isolated tibial cohort did. | Isolated tibial revision for aseptic tibial loosening has statistically similar clinical and radiological outcomes at a median follow- up of 3.5 years, when compared with full component revision. Femoral component revision for isolated tibial loosening can frequently be avoided provided adequate ligamentous stability can be obtained. | Single- vs full- component revision |
| Martin-Hernandez | 2017 | Mid-term results for metaphyseal sleeves in revision knee surgery | Knee Surgery, Sports Traumatology, Arthroscopy | Spain | Prospective case series | IV | 134 | To evaluate the mid-term results of radiological and clinical outcomes, for metaphyseal sleeves in type 1B and 2 defects of the tibia and femur in rTKA | Aseptic loosening 90%, Septic loosening 10%, Fracture 1% | 6 | rTKA with metaphyseal sleeves, in combination with uncemented stems and varus–valgus constrained components with rotating platform | Re-revision, Multiple PROMs, Surgical complications, Radiographic assessment | All clinical scores improved significantly in all patients. Complications included three cases of end-of-stem pain at tibial side and two revisions due to septic loosening in the first post-operative year. No aseptic loosening was reported. | The use of metaphyseal sleeves, in combination with uncemented stems and varus–valgus constrained components with rotating platform, has shown excellent mid-term results allowing osseous ingrowth with no evidence of osteolysis or displacement. | Zone 2 fixation devices |
| Meding | 2026 | Third-Generation Flexible Metaphyseal Cones in Revision Total Knee Arthroplasty: Minimum Two-Year Follow-up | Journal of Arthroplasty | United States | Case series | IV | 200 | To evaluate the early outcomes of flexible metaphyseal cones used to treat severe bone loss in rTKA | Instability 45%, Aseptic loosening 28%, Infection 14%, Osteolysis 10%, Arthrofibrosis 4% | 3 | rTKA with flexible metaphyseal cone (FMC) | Implant survivorship, Intraoperative fracture, Radiographic assessment | No radiolucent lines were identified around the FMCs. Survivorship was 98% for any revision, 99% for aseptic revision, and 100% for aseptic loosening. Excluding infection, all remaining FMCs (240 cones) demonstrated evidence of stable fixation. There were two intraoperative fractures (1%) that occurred | The FMCs provide stable fixation at early follow-up, and their ability to deform during impaction appears advantageous with a low rate of intraoperative fracture | Zone 2 fixation devices |
| Mills* | 2022 | No difference in long-term micromotion between fully cemented and hybrid fixation in revision total knee arthroplasty: a randomized controlled trial | The Bone & Joint Journal | Netherlands | Randomised controlled trial | II | 19 | To compare the migration patterns, clinical outcomes, adverse events, and re-revisions of cemented vs hybrid fixed stems in rTKA at ten year postoperatively | Not clearly defined; heterogeneous | 10 | Cemented vs press-fit stems | KSS, KOOS, VAS scores, Radiostereometric analysis, Adverse events | At radiostereometric analysis, there was no significant difference in femoral translation or rotation or tibial component translation or rotation, between the two groups. There were no differences in clinical scores. There was only one re-revision, in the hybrid group (infection). | These results show good long- term fixation with no difference in micromotion and clinical outcome between fully cemented and hybrid fixation in rTKA, which builds on earlier short- to mid- term results | Cemented vs hybrid fixation |
| Nelson | 2015 | Stemmed femoral implants show lower failure rates in revision total knee arthroplasty | The Knee | United States | Retrospective comparative study | III | 130 | To determine whether non-stemmed femoral implants are associated with a higher re-revision rate compared with stemmed femoral implants during rTKA | Infection and fracture excluded, otherwise not clearly defined | 2.0 (minimum) | rTKA with vs without stemmed femoral implants | Re-revision, Re-operation | Revisions performed without femoral stems failed more often than revisions with femoral stems(44% vs 9%, p =0.001), despite more severe pre-operative bone loss in groups that were revised with stems (p=0.05) | Femoral stems should be used routinely in procedures where a femoral implant is revised following a prior total knee arthroplasty | Cemented vs hybrid fixation |
| Nikolaus | 2016 | Factors Associated With 20-Year Cumulative Risk of Infection After Aseptic Index Revision Total Knee Arthroplasty | Journal of Arthroplasty | United States | Case series | IV | 1802 | To calculate the cumulative risk of periprosthetic joint infection (PJI) after aseptic index knee revisions and to identify the surgical, perioperative, and medical comorbidity risk factors associated with deep infection | Infection excluded, otherwise not clearly defined | 9.9 | Aseptic rTKA. Risk factors for PJI evaluated incl. use of constrained/hinged implant | Re-operation, Acquired medical comorbidities | Male gender, use of constrained implants, increased operative times, increased Charlson Comorbidity Index, and a history of liver disease were all significantly associated with PJI. | The risk of infection after revision TKA is higher than that seen after primary TKA, even with revisions that were performed for aseptic reasons. Further studies are warranted to not only identify these at-risk patients, but also to help determine how to minimize these risks of infection as much as possible | Constraint |
| Nwankwo | 2026 | Single- versus Dual-Component Revision Following Total Knee Arthroplasty Failure for Isolated Tibial or Femoral Loosening | Journal of Arthroplasty | United States | Retrospective comparative study | III | 288 | To examine outcomes between single- versus dual-component revision for isolated component loosening in TKA | Isolated tibial or femoral component loosening 100% | 4.5 | Single- versus full-component rTKA | Implant survivorship, Re-revision, 90-day complications, ROM, PROMs | Survivorship analysis showed comparable revision rates between both groups (P = 0.615). There were no differences found between the overall rerevision rate for single-component revision (11.2%, 16 of 134) and dual-component revision (9.7%, 15 of 154). The 90-day complications were similarly low in both groups (P > 0.05). Both cohorts demonstrated improvements in ROM and PROMs postoperatively, with no significant difference in PROMs (P > 0.05) | In patients who had isolated femoral or tibial loosening, single- and dual-component revision TKA offer comparable survivorship and functional outcomes. These findings support the use of surgeon discretion | Single- vs full-component revision |
| Onorato | 2025 | Stem fixation techniques in revision total knee arthroplasty: A systematic review and meta-analysis | Journal of Experimental Orthopaedics | United States | Systematic review | II | 1352 | To compare the clinical and radiological outcomes of patients undergoing rTKA using uncemented press‐fit stems (hybrid fixation) versus cemented stems (cemented fixation). | Not clearly defined; heterogeneous | 3.7 (minimum) | Cemented vs hybrid fixation | Implant survivorship, Re-operation, KSS, Radiographic assessment | Survival rates of hybrid and cemented fixations were comparable, with a significant trend favouring hybrid fixation (p=0.04). Meta‐analysis indicated better results with hybrid fixation, although not statistically significant (KSS functional p = 0.15; KSS clinical p = 0.5). High heterogeneity was observed due to variations in patient characteristics and surgical strategies. | Both hybrid and cemented fixation techniques achieve satisfactory clinical results in rTKA, with hybrid fixation demonstrating an overall lower failure rate. Further high-quality randomised trials are needed to refine these results and optimise fixation strategies to improve patient outcomes. | Cemented vs hybrid fixation |
| Parish | 2025 | A Comparison of Isolated Tibial Insert Exchanges for Global Instability to Full Revisions for Flexion Instability in Revision Total Knee Arthroplasty | Arthroplasty Today | United States | Retrospective comparative study | III | 172 | To compare outcomes of patients undergoing isolated polyethylene exchange versus full-component revision for instability | Instability 100% | 2.2 | Full-component revision versus isolated polyethylene exchange | UCLA, KOOS, KSS | While not statistically significant, a greater percentage of FCR patients met the minimal clinically important difference threshold for the Knee Injury and Osteoarthritis Outcome Score for Joint Replacement (69 vs 38%; P =.11); while there was no difference in minimal clinically important difference between FCR and ITIE in other patient-reported outcome measures (P ≥.28). Patients were “very satisfied or satisfied" in 58% of the ITIE group vs 56% of the FCR group (P =.51). | ITIE can be successful after revision TKA given the specific indication of symmetric global instability with fixed and well-positioned components. However, FCR is preferred in the case of flexion instability and the surgeon must be vigilant in their instability etiology | Isolated polyethylene exchange |
| Park | 2019 | Factors affecting the choice of constrained prostheses when performing revision total knee arthroplasty | International Orthopaedics | South Korea | Case series | IV | 274 | To assess the levels of prosthetic constraint chosen during revision total knee arthroplasty (TKA) and to identify factors influencing the choice of a constrained prosthesis | Polyethylene wear 73%, Infection 10%, Aseptic loosening 7%, Fracture 7%, Instability 3%, Stiffness 1% | 7.2 | Constrained (CCK/Hinge) vs Less-constrained (CR/PS) implants at rTKA | KSS, WOMAC, ROM, Radiographic assessment | The clinical score and ROM at the last follow-up after revision TKA were better in the less-constrained group, but there was no significant difference in the change of any knee or function score, the WOMAC score, or the ROM, between the two groups | Consideration of various factors including the cause of revision TKA and changes in the joint line height and Insall-Salvati ratio will aid the TKA surgeon in selecting prostheses with appropriate constraints when performing rTKA | Constraint |
| Patel | 2004 | The fate of augments to treat type-2 bone defects in revision knee arthroplasty | Journal of Bone & Joint Surgery | Canada | Prospective case series | IV | 102 | To report the five- to ten-year results on the specific use of metal augmentation devices with knee revision implants for Anderson Orthopaedic Research Institute (AORI) type-2 bone defects | Wear and osteolysis 27%, Infection 25%, Aseptic loosening 21%, Patellofemoral complications 12%, Instability 11%, Polyethylene wear 10%, Fracture 2%, Pain 2% | 7 | rTKA in patients with AORI type 2 defects treated with augments and stems | Re-revision, Implant survivorship, KSS, Radiographic assessment | The presence of nonprogressive radiolucent lines around the augment in 14% of knees was not associated with poorer knee scores, the range of movement, survival of the component or the type of insert which was used (p > 0.05). The survival of the components was 92 ± 0.03% at 11 years (95% CI, 10.3 to 11.2). | The authors recommend the use of modular augmentation devices to treat type-2 defects in revision knee surgery | Other- Zone 1 augmentation |
| Piuzzi | 2024 | Short-term Comparison of Survivorship and Functional Outcomes for Metaphyseal Cones with Short and Long Stems in Revision Total Knee Arthroplasty | The Journal of Knee Surgery | United States | Retrospective comparative study | III | 179 | To compare the short-term survivorships and functional outcomes for use of a short or long stem with a metaphyseal cone | Not defined; heterogeneous | 2 | rTKA using metaphyseal cones and short vs long stem | Re-revision, Implant survivorship, Multiple PROMs | Revision-free survivorship for the femoral or tibial component was 100% for long stems and 98.2% for short stems at 1 and 2 years, respectively (p=0.6330).The two revisions in the short group were for infection, thus the survivorship for aseptic loosening was 100% at 2 years for both cohorts. There were no significant differences in preoperative or postoperative PROMs | Highly porous metaphyseal cones provided rTKA with excellent early survivorship and similar PROMs whether a short or long stem was used. Additional studies will be needed to discern longer term differences | Zone 2 fixation devices; Cemented vs hybrid fixation |
| Puri | 2023 | Outcomes After Revision Total Knee Arthroplasty From a Specific, Now-Recalled Implant System | Journal of Arthroplasty | United States | Case series | IV | 202 | To report the early outcomes of aseptic revision at our institution, compare survivorship following isolated polyethylene exchange versus revisions in which the femoral or tibial component was revised, compare survivorship after revision with the recalled components versus other implant systems, and to use a Cox proportional hazards regression model to ascertain risk factors for re-revision | Aseptic loosening 59%, Instability 27%, Polymeric wear/osteolysis 13% | 4 | Aseptic rTKA of a primary Exactech TKA, outcomes compared in isolated polyethylene exchange vs component revision and Exactech revisions vs non-Exactech implants | Re-revision, Implant survivorship, Re-revision implant use | At 2 and 5 years, survivorship free from all-cause rerevision was 89 and 76% in the polyethylene exchange cohort versus 92 and 84% in the component revision cohort (p=0.5). At 2 and 5 years, survivorship in revision with components from the same manufacturer was 89 and 80% compared to 95 and 86% in revision with components from a different manufacturer (p=0.2) | In this series of aseptic revision TKAs performed on a now-recalled implant system, survivorship free from rerevision was lower than expected when components from the same manufacturer were utilised, but comparable to contemporary reports when both the components were revised with an alternative implant system | Isolated polyethylene exchange |
| Quinn | 2022 | Clinical Outcomes Following Revision Total Knee Arthroplasty: Minimum 2-Year Follow-up | Clinics in Orthopaedic Surgery | Australia | Case series | IV | 153 | To investigate and report the mid- to long-term outcomes in patients undergoing rTKA performed by a single surgeon using a single prosthesis (PFC, DePuy Synthes) at a single institution | Aseptic loosening 33%, Infection 22%, Instability 9%, Progression of OA 8%, Pain 7%, Stiffness 3%, Other 16% | 6.5 | rTKA using a single system. Factors associated with clinical outcome evaluated | Re-revision, OKS, ROM, Patient satisfaction, Surgical complications, Re-admission | This cohort demonstrated a 93.5% survival rate and an 85% satisfaction rate at a mean of 6.5 years postoperatively. Mean ROM improved from 100° (range, 5°–145°) to 112° (range, 35°–135°) (p < 0.001). The mean OKS was 39.25 (range, 14–48). The factors associated with improved postoperative outcomes included male sex, fewer previous revision total knee arthroplasty procedures, increased preoperative ROM, and receiving a less constrained implant | Although revision total knee arthroplasty is a challenging and complex aspect of arthroplasty surgery, high patient satisfaction and good functional outcomes can be achieved for the majority of patients | Constraint |
| Ritter | 2004 | Predicting range of motion after revision total knee arthroplasty: clustering and log-linear regression analyses | Journal of Arthroplasty | United States | Case series | IV | 355 | To determine which variables affect range of motion following revision total knee arthroplasty | Not defined; heterogeneous | 2.4 | rTKA. Factors associated with postoperative ROM evaluated | Post-operative flexion and extension | The mean preoperative and postoperative f lexion were 100.5° and 104.6°. Low preoperative ( 103°) and intraoperative flexion ( 117°), young age ( 44 years), and constrained and hinged prosthesis types were associated with diminished flexion | A patient with a low preoperative flexion, a low intraoperative flexion, a young age, and a hinged or constrained prosthesis would have the worst predicted postoperative flexion | Constraint |
| Roach | 2021 | Aseptic Loosening of Porous Metaphyseal Sleeves and Tantalum Cones in Revision Total Knee Arthroplasty: A Systematic Review | The Journal of Knee Surgery | United States | Systematic review | II | 1753 | To examine and review the current literature regarding sleeves and tantalum cones as TKA revision implants with particular attention paid to the survivorship, incidence of aseptic loosening, and reported reoperations. | Not defined; heterogeneous | Mean range 2.0-6.0 | rTKA with metaphyseal cones or sleeves | Re-operation, Rate of aseptic loosening | In 12 studies investigating metaphyseal sleeves, the overall rate of reoperation was 110/1,133 (9.7%) and the total rate of aseptic loosening per sleeve was 13/1,617(0.8%). In the 15 studies on tantalum cones, the overall rate of reoperation was 116/620 (18.7%), and the overall rate of aseptic loosening per cone was 12/701 (1.7%) | Rates of aseptic loosening of the two implants were found to be similar, while the rate of reoperation was nearly double in revision TKAs utilising tantalum cones. Variability in the selected studies and the likely multifactorial nature of failure do not allow for any definitive conclusions to be made | Zone 2 fixation devices |
| Ruelos | 2026 | Rotating-Hinge Revision Total Knee Arthroplasty for Arthrofibrosis Improves Motion Without Increasing Complications: A Systematic Review and Meta-Analysis | Journal of Arthroplasty | United States | Systematic review | II | 224 | To evaluate pooled ROM improvement following rotating hinge revision for stiffness and to compare ROM outcomes between rotating hinge and non-hinged revision constructs | Stiffness/arthrofibrosis 100% | 4.6 | Rotating hinge vs non-hinged revision constructs | ROM, Postoperative complications, Reoperations | The rotating hinge group resulted in a significant pooled improvement in ROM of 42.2deg (95% CI, 32.3 to 52.1; P < 0.0001). Comparative analysis of four studies (224 RH versus 108 NHR revisions) demonstrated significantly greater ROM improvement with rotating hinge implants, with a pooled between-group difference of 17.5deg favouring RH constructs (95% CI, 6.6 to 28.4deg; p=0.002) | Compared with nonhinged constrained revision constructs, RH implants yield significantly greater gains in motion without an apparent increase in complications or reoperation | Constraint |
| Sapountzis | 2023 | The Role of Constraint in Revision Total Knee Replacement for Instability: Full Component Revision Vs Isolated Polyethylene Exchange in Selected Patients | Arthroplasty Today | United States | Retrospective comparative study | III | 117 | To determine (1) whether isolated polyethylene exchange results in similar re-revision frequency to component revision in select patients with symptomatic instability and (2) the effect of increasing constraint on the outcome | Instability 100% | 3 | Isolated polyethylene exchange vs full component revision; constraint level | Re-revision, Multiple PROMs, ROM | The re-revision rate was 18%, with no statistical difference between component and isolated polyethylene exchange cohorts. Cases where level of constraint increased due to revision, a significantly lower rate of re-revision was detected (9 of 77) (12%) than in cases where constraint did not increase (12 of 39) (31%) (p=0.012). | Re-revision occurred at similar frequencies 2 years after isolated polyethylene exchange or component revision for total knee arthroplasty instability. For component revision, increased constraint was associated with significantly fewer re-revisions | Isolated polyethylene exchange; Constraint |
| Shen | 2014 | Revision knee arthroplasty for bone loss: choosing the right degree of constraint | Journal of Arthroplasty | United States | Retrospective comparative study | III | 475 | To determine the difference in the outcome of revision total knee arthroplasty in the setting of bone defects when conducted with different degrees of constrained prostheses | Aseptic indications 72%, Infection 28% | 7.4 | Constraint level; PS, Unlinked constrained, or Hinge | KSS, SF-36, WOMAC, Radiographic assessment, Surgical complications | PS yielded superior knee scores in AORI Type I patients (p=0.05), unlinked constrained in Type II and III aseptic patients (p=0.05), and a hinge was preferred in septic Type II or III knees (p=0.05) | Our results suggest that prostheses witha lower degree of constraint provide more superior functional outcome.The only exception is that patients undergoing revision arthroplasty for infection who have type II or III bone defect may benefit from the use of linked constraint prosthesis | Constraint |
| Sheng | 2006 | Revision total knee arthroplasty: 1990 through 2002. A review of the Finnish arthroplasty registry | Journal of Bone & Joint Surgery | Finland | Case series | IV | 2637 | To present the results of revision total knee replacements and describe predictors of survival of those replacements, with repeat revision as the end point | Aseptic loosening 33%, Patellar complications 32%, Other 35% | Not stated | rTKA. Predictors of implant survival evaluated. | Implant survivorship | The survival rate following the revision total knee arthroplasties was 95% (95% confidence interval, 94% to 96%) at two years (1874 knees), 89% (95% confidence interval, 88% to 90%) at five years (944 knees), and 79% (95% confidence interval, 78% to 81%) at ten years (141 knees). Multivariate regression analysis showed the most significant predictors of prosthetic survival to be the age of the patient and the life in service of the primary total knee replacement. Cement fixation (p < 0.005) and bone-grafting (p = 0.05) improved prosthetic survival, whereas hybrid fixation did not differ significantly from cementless fixation | An age greater than seventy years, revision five years or more after the primary arthroplasty, and absence of patellar subluxation are positive indicators of survival of a revision total knee replacement. | Cemented vs hybrid fixation |
| Sheridan | 2021 | Hybrid stems are superior to cemented stems in revision total knee arthroplasty: a systematic review and meta-analysis of recent comparative studies | European Journal of Orthopaedic Surgery & Traumatology | Canada | Systematic review | II | 1179 | To explore the optimal fixation method for stems in revision TKA using data from recent, well-designed comparative studies | Not clearly defined; heterogeneous | 5.3 | Cemented vs hybrid fixation | Re-revision, Aseptic loosening, Multiple PROMs, Radiographic assessments | There was a significantly lower failure rate for hybrid stems when compared to cemented stems (p=0.006) (OR 0.61, 95% CI 0.42–0.87). There was a trend toward superior hybrid performance for all other outcome measures including all-cause re-revision, aseptic re-revision and radiographic failure. | The authors recommend the use, where possible, of hybrid stems in revision TKA. | Cemented vs hybrid fixation |
| Sheth | 2026 | Isolated versus Full-Component Revision Total Knee Arthroplasty for Aseptic Loosening: Systematic Review and Meta-Analysis | Journal of Arthroplasty | United States | Systematic review | II | 3634 | To compare outcomes of isolated versus full-component revision TKA for aseptic loosening, with particular focus on complications, implant survivorship, and functional results | Aseptic loosening 100% | 0.3-8 | Single- versus full-component rTKA | Re-revision, operative time, blood loss, ROM, length of stay, patient satisfaction | Isolated and full component rTKA demonstrated comparable rates of re-revision for all indications (odds ratio (OR): 1.36, 95% confidence interval (CI) 0.96 to 1.95, p=0.094). The isolated component rTKA showed significantly reduced operative time (Standardized Mean Difference=2.85, 95% CI 1.47 to 4.23, P < 0.001), and two studies reported lower intraoperative blood loss, while postoperative range of motion was similar between approaches | Isolated component revision demonstrated outcomes comparable to full component revision. These findings support the idea that when components are well-fixed and compatible, isolated component revision may serve as a less morbid option for rTKA | Single- vs full-component revision |
| Shichman | 2023 | The impact of posterior-stabilized vs. constrained polyethylene liners in revision total knee arthroplasty | Archives of Orthopaedic & Trauma Surgery | United States | Retrospective comparative study | III | 314 | To compare implant survivorship, complications, and clinical outcomes between patients who underwent rTKA with either PS or VVC liners at a minimum of 2-year follow-up | Aseptic loosening 33%, Instability 26%, Arthrofibrosis 14%, Infection 10%, Component malpositioning 4%, Other 6% | 3.6 | Posterior stabilised vs varus valgus constrained liners in rTKA | Re-revision, Implant survivorship, ROM, Radiographic assessment, Length of stay | The proportion of patients undergoing re-revision did not significantly differ (19.1% vs. 18.7%, p = 0.929). In subgroup analysis of re-revision causes, the VVC cohort had superior survival from re-revision due to instability compared to the PS cohort (97.8% vs. 89.4%, p = 0.003). Freedom from re-revision due to aseptic loosening did not significantly differ between groups (85.2% vs. 78.8%, p = 0.436). Improvements in range of motion (ROM) from preoperative to latest follow-up were similar as well. | PS and VVC liners confer similar survivorship, complication rates, and overall knee ROM in rTKA. Future studies with longer follow-up are warranted to better determine significant differences in clinical outcomes between the two bearing options | Constraint |
| Shichman | 2023 | Comparison of Aseptic Partial- and Full-Component Revision Total Knee Arthroplasty | Journal of Arthroplasty | United States | Retrospective comparative study | III | 293 | To compare the outcomes of partial versus full-component exchange in aseptic revision TKA | Aseptic loosening 46%, Instability 22%, Arthrofibrosis 15%, Component malpositioning 5%, Fracture 3%, Liner wear 3%, Implant failure 3%, Extensor mechanism 3%, Other 1% | 4.2 | Partial component revision vs total component revision | Re-revision, Implant survivorship, Multiple PROMs, ROM | Re-revision rates did not significantly differ between groups (P-rTKA=11.8% vs F-rTKA=16.1%, p=0.358). There were no differences in PROMs. | Compared to F-rTKA, P-rTKA yielded similar functional outcomes and implant survivorship with shorter surgical time. When indications and component compatibility allow for such a procedure, surgeons can expect good outcomes when performing P-rTKA | Single- vs full-component revision |
| Shichman | 2023 | Mid term outcomes of a novel metaphyseal porous titanium cone in revision total knee arthroplasty | Journal of Clinical Orthopaedics and Trauma | United States | Case series | IV | 104 | To examine mid-term functional and radiographic outcomes in patients undergoing rTKA utilising a newly designed metaphyseal cone system | Aseptic loosening 35%, Infection 22%, Instability 17%, Other 26% | 2.7 | rTKA with metaphyseal porous titanium cone | Re-revision, Implant survivorship, ROM, Radiographic assessment | Thirteen rTKA underwent re-revision surgery: 3 for acute PJI, 4 for chronic PJI, 5 for instability, and 1 for mechanical failure of a hinged system. All-cause re-operation free survivorship was 87.5 % (91/104), and all-cause cone implant survivorship was 96.09 % (123/128 cones) at 2-year follow-up. | This study demonstrates excellent mid-term outcomes of a novel porous, titanium metaphyseal cone in patients with large bone defects undergoing complex revision TKA. | Zone 2 fixation devices |
| Shield | 2019 | Ignore the Patella in Revision Total Knee Surgery: A Minimum 5-Year Follow-Up With Patella Component Retention | Journal of Arthroplasty | United States | Case series | IV | 147 | To report results and patient outcomes after retention of a well-fixed, all-polyethylene patella component in a consecutive series of patients undergoing revision by a single surgeon at a single institution | Aseptic indications 100%; infection excluded | 5.0 (minimum) | Aseptic rTKA with retention of the patella component | Re-operation, KSS, ROM, Radiographic assessment | At final follow-up, there were no reoperations on any patella and none were at risk of failure. There were 6 knees with a lateral patella tilt beyond 10 , but none were subluxed. Knee Society Scores averaged 85 ± 17.2 points at final follow-up. | At midterm follow-up in this group of RTKA where the patella was not revised, we identified no subsequent failures of the patella. This is despite the presence of mild patella polyethylene wear and mismatched shapes in several knees. | Other- management of patella at tibiofemoral rTKA |
| Song | 2026 | Thick metal augmentation is associated with inferior implant survival compared with structural allografts in Anderson Orthopaedic Research Institute Type 2A uncontained medial tibial bone loss | Orthopaedics & traumatology, surgery & research | South Korea | Retrospective comparative study | III | 144 | To evaluate tibial implant survival in revision TKA with either thick metal augments (≥10 mm thickness) or structural allografts for AORI type 2A uncontained medial tibial loss | All first-time elective aseptic rTKA procedures, fracture/infection excluded, otherwise not clearly defined | 6.0-8.2 | rTKA with either thick metal augments (≥10 mm thickness) or structural allografts for AORI type 2A uncontained medial tibial loss | Radiographic assessment (tibial component loosening), Implant survivorship | The incidence of tibial component loosening was 14.3% (10/70) the metal augments group and 8.1% (6/74) in the allograft group (p=0.294). The 5-, 10-, and 15-year survival rates were 87.2%, 83.6%, and 83.6%, in the metal augments group, and 97.0%, 97.0%, and 92.6% in the allograft group, respectively (p=0.015) | Thick metal augmentation may be associated with inferior implant survival compared to structural allografts for AORI type 2A uncontained medial tibial bone loss. Stress shielding–induced bone resorption should be carefully considered when thick metal augments are used | Other- augments |
| Souto | 2026 | Risk factors for aseptic loosening after revision total knee replacement (rTKR): a component-specific analysis of femoral and tibial reconstruction | The Knee | Portugal | Case-control study | III | 215 | To evaluate the association between the use of sleeves and augments and the risk of re-revision following rTKA performed for aseptic loosening | Aseptic loosening 100% | Minimum 2 years | rTKA for aseptic loosening. Implant-related factors compared between those undergoing re-revision and those who did not | Re-revision | Tibial augments were significantly more frequent in cases requiring re-revision. Metaphyseal sleeves were less frequently used in cases requiring rerevision, although this difference did not reach statistical significance | Tibial augments were associated with a higher rate of re-revision following rTKA, likely reflecting increased reconstruction. Metaphyseal sleeves showed lower failure rates, although this did not reach statistical significance | Other- augments, Zone 2 fixation devices |
| Stevens | 2019 | Survival and functional outcome of revision total knee arthroplasty with a total stabilizer knee system: minimum 5 years of follow-up | European Journal of Orthopaedic Surgery & Traumatology | United Kingdom | Case series | IV | 100 | To determine midterm allcause survivorship of the Stryker Triathlon total stabilizer knee revision system. The secondary aims were to assess midterm functional outcome, patient satisfaction and radiographic outcome | Not defined; heterogeneous | 7.2 | rTKA with Triathlon Total Stabiliser knee system | Implant survivorship, Multiple PROMs, Patient satisfaction, Radiographic assessment | The all-cause survival rate at 5 years was 89.0% [95% confidence interval (CI) 87.3 to 90.7]. The mean OKS was 27 (SD 11.9, range 0 to 46), FJS was 32.3 (SD 30.4, range 0 to 100), SF-12 physical component summary was 40.6 (SD 17.6, range 23.9 to 67.1), and mental component summary was 48.3 (SD 15.5, range 23.9 to 69.1). Reported patient satisfaction in patients who were not re-revised was 82% | The midterm survivorship of cemented Stryker Triathlon TS knee revision for all-cause mode of failure is good to excellent; however, future follow-up is required to ensure this survivorship is observed into the long term. | Constraint; Cemented vs hybrid fixation |
| Stockwell | 2019 | Clinical and radiographic outcomes of a hybrid fixation revision total knee arthroplasty system at short to mid-term follow-up | The Knee | Canada | Case series | IV | 234 | To report the short and mid-term results of a single hybrid fixation rTKA system | Aseptic loosening 31%, Stiffness/pain 23%, Instability/Component wear 21%, Infection 18%, Fracture/dislocation 3%, Other 4% | 4.9 | rTKA with hybrid fixation utilising the Legion Revision Total Knee Arthroplasty (Smith&Nephew) | Implant survivroship, OKS, Patient satisfaction, Radiographic assessment | Aseptic survivorship at one year, two years, and five years was 100%, 100%, and 99.1%, respectively. Any-cause survival at one, two, and five years was 99.6%, 98.7%, and 92.3%, respectively. OKS improved from pre-op (average 18.8) to one year (average 31.7), two years (average 30.7), and mid-term (average 30.6) follow-up (p<0.001 for all). At all intervals, patient satisfaction exceeded 70% | The survivorship, clinical, and radiographic outcomes of the single rTKA system studied are equivalent or superior to other hybrid fixation rTKA systems reviewed in the literature at similar follow-up intervals | Cemented vs hybrid fixation |
| Stroobant | 2023 | Condylar constrained and rotating hinged implants in revision knee arthroplasty show similar survivorship and clinical outcome: a systematic review and meta-analysis | Knee Surgery, Sports Traumatology, Arthroscopy | Belgium | Systematic review | II | 4555 | To compare the survival rates of a CCK and an RHK, compare the differences in clinical outcomes between these two types of implants and evaluate if there is a higher reoperation rate with RHK compared to CCK | Aseptic loosening 43.1%, Infection 24%, Instability 23%, Arthrofibrosis 5%, Fracture 3%, Malalignment 2% | 2.0 (minimum) | Condylar constrained vs rotating hinge implants in rTKA | Implant survivorship, KSS, ROM | Survival did not differ between RHK and CCK implants (p = 0.6058), with, respectively, 91.6% and 89.8% survival after 5 years. Both groups demonstrated similar KSS scores and postoperative ROM. | This meta-analysis revealed that both survivorship and clinical outcome are similar for CCK and RHK patients for whom both designs are technically suitable and indicated. | Constraint |
| Stroobant | 2025 | Instability after total knee arthroplasty: A systematic review and meta-analysis of treatment options and outcomes | Knee Surgery, Sports Traumatology, Arthroscopy | Belgium | Systematic review | II | 4269 | To evaluate the outcomes of conservative management, isolated polyethylene exchange (PE), and rTKA as treatments for post‐TKA instability | Instability 100% | Range 2.7-8.3 | Conservative management, isolated polyethlene exchange or rTKA for the management of instability post-TKA | PROMs, Reoperation rates, Re-revision, Implant survivorship | Conservative treatment, primary physiotherapy, yielded inconsistent results. Isolated PE exchange showed poor outcomes, with recurrent instability and subsequent revision occurring in 6.5%–18.5% of cases. However, in carefully selected patients, outcomes comparable to rTKA were observed | When guided by appropriate clinical indications, conservative management, isolated PE exchange, and revision surgery can yield favourable outcomes in the treatment of post‐TKA instability. Instability remains the leading cause of rerevision, highlighting the need for higher prosthetic constraints when flexion‐extension gap balancing cannot be achieved | Isolated polyethylene exchange |
| Tegethoff | 2022 | Isolated Liner Revision for Total Knee Arthroplasty Instability: A Road That Should Remain Less Taken | The Journal of Knee Surgery | United States | Retrospective comparative study | III | 146 | To assess the relative success of isolated polyethylene exchange compared with revision of both tibial and femoral components for the treatment of isolated prosthetic knee instability | Instability 100% | 2.0 (minimum) | Isolated polyethylene exchange vs revision of femoral and tibial components | Re-operation, Re-revision | Patients undergoing isolated polyethylene exchange were more likely to undergo reoperation (60.0 vs.17.5%, p=0.001), component revision surgery (45.0 vs 8.7%, p=0.002), and component revision within 2 years (30.0 vs 1.6%, p<0.0001). Differences in 90-dayreoperation(p=0.14) and revision >2years (p=0.19) were not significant. Re-operation for instability (30.0 vs 4.0%, p<0.001) and infection (20.0 vs 1.6%, p<0.01) were both higher in the isolated polyehtylene exchange group | Isolated polyethylene exchange does not provide consistent benefits for patients undergoing TKA revision for instability. Considerations for lower immediate postoperative morbidity and cost need to be carefully measured against long-term consequences of reoperation, delayed component revision, and increased long-term costs of multiple surgical procedures | Isolated polyethylene exchange |
| Tetreault | 2021 | Isolated tibial insert exchange in revision total knee arthroplasty : reliable and durable for wear; less so for instability, insert fracture/dissociation, or stiffness | The Bone & Joint Journal | United States | Case series | IV | 270 | To re- evaluate the clinical outcomes of selective isolated tibial insert exchange in aseptic rTKA, including a larger cohort of patients with longer- term follow- up. | Instability 55%, Polyethylene wear 39%, Insert fracture/dissociation 5%, Stiffness 1% | 7 | Aseptic rTKA with isolated tibial insert exchange | Re-revision, Implant survivorship, KSS, Factors associated with re-revision | Survivorship free of any re- revision was 68% at ten years. For the indication of insert wear, survivorship free of any re- revision at ten years was 74%. Re- revisions were more frequent for index diagnoses other than wear (hazard ratio (HR) 1.9; p = 0.013), with ten- year survivorships of 69% for instability and 37% for insert fracture/dissociation. Mean Knee Society Scores improved from 54 (0 to 94) preoperatively to 77 (38 to 94) at ten years. | The best results were for polyethylene wear. For other diagnoses, the re- revision rate was higher and the failure mode was most commonly recurrence of the original indication for the revision TKA | Isolated polyethylene exchange |
| Theil | 2022 | Good to excellent long-term survival of a single-design condylar constrained knee arthroplasty for primary and revision surgery | Knee Surgery, Sports Traumatology, Arthroscopy | Germany | Case series | IV | 143 | To determine the long-term survivorship, functional outcomes of a single-design condylar constrained (CCK) TKA in primary and revision cases as well as to assess speciic risk factors for failure | Aseptic loosening 41%, Instability 25%, Exchange for PJI 25%, Malpositioning 5%, Fracture 3% | 11.8 | rTKA or complex primary TKA using a single design condylar constrained knee system (Genesis CCK, Smith&Nephew) | Re-revision, Implant survivorship, OKS | The implant survival was 86.4% after ive, 85.5% after ten and 79.8% at 15 years. Patients who underwent primary TKA had a higher revision-free implant survivorship compared to revision TKA at 15 years (100% vs. 76%, p = 0.036). The main cause for re-revision was infection in 10% of all revision TKA performed with the CCK design included, while no case was revised for instability | CCK implants are associated with excellent long-term survival when used in primary TKA; however, survival was worse when used during revision TKA. While instability and aseptic loosening were rare, infection remains a major concern. | Constraint |
| Thever | 2024 | A systematic review and meta-analysis of hybrid vs. cemented stems - which method is more optimal for revision total knee arthroplasty? | BMC Musculoskeletal Disorders | Singapore | Systematic review | II | 1555 | To provide an updated insight and combine existing studies in the literature to explore and evaluate the optimal fixation method for stems in revision TKA, and provide clinical guidance based on the existing literature | Heterogeneous; proportions not defined | 2.0 (minimum) | Cemented vs hybrid fixation | Re-revision, Multiple PROMS, Radiographic assessments | There was no statistically significant difference in radiological failure and loosening (OR=0.79, CI 0.37–1.66, I2= 29%, p=0.22), all causes of re-revision (OR =1.03, CI 0.73–1.44, I2=0%, p=0.56) and aseptic revision (OR=0.74, CI 0.27–2.02, I2=0%, p 0.41) between cemented and hybrid techniques. Functional and pain outcomes compared between the two fixation techniques were largely similar | Despite a trend favouring hybrid stems in revision TKA, current evidence revealed that radiological outcomes and re-revision rates are largely similar between cemented and hybrid fixation techniques | Cemented vs hybrid fixation |
| Tracey | 2021 | Clinical Outcomes in Isolated Tibial Revision With Cruciate Retaining Total Knee Arthroplasty | Journal of Arthroplasty | United States | Case series | IV | 135 | To determine the results of isolated tibial revisions in cruciate retaining TKA | Aseptic loosening 100% | 5.1 | Isolated tibial component revision | Re-revision, Implant survivorship, KSS, KOOS, Radiographic assessment | There were six (4.4%) repeat revisions: three for periprosthetic infection (2.2%), two for instability (1.5%), and one for a fractured tibial stem (0.7%). KSS and KOOS scores improved postoperatively (p<0.001). Survivorship free of repeat revision for any cause was 93.3% at 5 years, and aseptic revision survivorship was 95.8% at 5 years. | In patients with isolated tibial loosening and a well-fixed and well-positioned CR femoral component, isolated tibial revision provides excellent early to midterm implant survivorship and clinical outcomes with a low risk of instability and recurrent tibial loosening | Single- vs full- component revision |
| Turnbull | 2019 | Gender and Preoperative Function Predict Physical Activity Levels After Revision Total Knee Arthroplasty | Journal of Arthroplasty | United Kingdom | Case series | IV | 112 | To examine factors influencing return to physical activity following revision TKA | Aseptic loosening 39%, Instability 25%, Infection 23%, Pain/stiffness 5%, Implant failure 3%, Polyethylene wear 2%, Other 3% | 3.9 | Tibiofemoral rTKA with semi-constrained or hinged implants | Re-operation, Multiple PROMs, Medical and surgical complications | Mean UCLA activity scores improved from preoperative levels (p<0.001): activity levels improved in 47% of patients with 58% engaging in moderate or more intensive activities (UCLA score 5). Indication, implant type, and patient body mass index did not affect functional outcome or satisfaction (p>0.05) | Although 90% of patients maintain activity levels following revision TKA, less than half increase levels and this is predicted by male sex and pre-revision activity level. | Constraint |
| Tyas | 2026 | Condylar-constrained prostheses are associated with favourable implant survival following first-time aseptic revision knee arthroplasty: An analysis from the National Joint Registry | Knee Surgery, Sports Traumatology, Arthroscopy | United Kingdom | Retrospective comparative study | III | 12950 | To compare implant survivorship and patient-relevant outcomes associated with the constraint level used at first-time aseptic rTKA | Aseptic loosening 52%, Malalignment 16%, Instability 16%, Stiffness 7%, Unexplained pain 5%, Component wear 3% | 7.4 | rTKA with unconstrained, posterior-stabilised, condylar-constrained or hinged implants | Re-revision, PROMs, 90-day mortality and complications, length of stay | CCK prostheses were associated with the lowest unadjusted 10‐year cumulative incidence of re-revision overall (9.2% [95% CI 8.4–10.1]) and in all subgroups. In the primary adjusted analysis, unconstrained (subdistribution hazard ratio [sHR] 1.46 [1.23–1.74], p < 0.001), PS (sHR 1.23 [1.04–1.44], p = 0.013) and hinged prostheses (sHR 1.66 [1.38–2.01], p < 0.001) were associated with higher re‐revision risk than CCK constructs | CCK constructs were consistently associated with favourable implant survivorship across a range of clinical scenarios. These findings provide reassurance that clinically indicated use of CCK constraint is not associated with compromised implant survival | Constraint |
| vanLaarhoven | 2022 | Superior Survival of Fully Cemented Fixation Compared to Hybrid Fixation in a Single Design Rotating Hinge Knee Implant | Journal of Arthroplasty | Netherlands | Retrospective comparative study | III | 275 | To compare the rate of aseptic loosening with fully cemented fixation compared to hybrid fixation in a single design rotating hinge knee implant | Instability 31%, Stiffness 28%, Septic 15%, Aseptic 13%, Malposition 11%, Fracture 2% | 7.3 | Primary or rTKA using the RT-PLUS rotating hinge knee implant (Smith&Nephew) with all-cemented vs hybrid fixation | Re-revision, Implant survivorship | In total, 24 components (16 hybrid femur, 2 fully cemented femur, 6 hybrid tibia; all revision procedures) in 19 patients were revised for aseptic loosening. Kaplan-Meier survivorship analysis showed superior survival rates of fully cemented components (femur 97.1%; tibia 100%) compared to hybrid fixated components (femur 89.5%; tibia 95.9%) at the 10-year follow-up. | Fully cemented fixation showed superior survival rates for aseptic loosening compared to hybrid fixation in a single design rotating hinge knee implant. A prior stemmed component appears to be a risk factor for aseptic loosening and the femoral component seems to be more prone to loosening | Cemented vs hybrid fixation; Constraint |
| Villa | 2025 | The Fate of Highly Porous Titanium Tibial Cones in Revision Total Knee Arthroplasty: A Multicenter 5-Year Minimum Follow-Up Study | Journal of Arthroplasty | United States | Retrospective comparative study | III | 151 | To assess the overall tibial cone survivorship rate after rTKA and to compare cone survivorship between septic and aseptic revisions | Aseptic 73%, Septic 27% | 5.7 | rTKA using Tritanium (Stryker) tibial metaphyseal cones | Re-revision, Implant survivorship, Radiographic assessment | Thirty patients (19.9%) underwent a subsequent total knee arthroplasty revision. At the latest follow-up, according to the Knee Society grading system, 115 (95.0%) of surviving rTKAs were deemed stable, three (2.5%) warranted close observation, and three (2.5%) were loose. the overall cone survivorship rate was 90.7% with no significant differences between survivorship rates of cones implanted during either septic (87.5%) or aseptic (91.9%) rTKAs (p=0.52) | Our data support the use of highly porous titanium tibial cones to improve metaphyseal fixation during rTKA even when there is a history of a knee infection. | Zone 2 fixation devices |
| Wang | 2016 | Fixation of stem in revision of total knee arthroplasty: cemented versus cementless-a meta-analysis | Knee Surgery, Sports Traumatology, Arthroscopy | Germany | Systematic review | II | 1770 | To compare implant survivorship following rTKA with cemented or cementless stem fixation | Not defined; heterogeneous | Mean range 3.2-10.2 | Cemented vs cementless stems | Re-operation, Radiographic failure, Aseptic loosening, Infection rate | There was a similar likelihood of failure for any reason (risk ratio, RR 0.97), general reoperation (RR 1.02), aseptic loosening (RR 1.0) and infection (RR 1.0) in cemented stem fixation group compared to cementless stem fixation group with follow-up <60 months. When follow-up period extend to more than 60 months, the same likelihood was observed | There was no significant difference in failure for any reason, reoperation, aseptic loosening and infection between revision TKA with cemented or cementless stem fixation. Based on the available literature, no superiority of any type of stem fixation was found. | Cemented vs hybrid fixation |
| Watters | 2017 | Porous-Coated Metaphyseal Sleeves for Severe Femoral and Tibial Bone Loss in Revision TKA | Journal of Arthroplasty | United States | Case series | IV | 116 | To evaluate the mid-term results of stepped, porous-coated metaphyseal sleeves in a large series of revision TKA operative procedures with severe bone loss | Aseptic loosening 25%, Infection 24%, Instability 18%, Polywear/Osteolysis 16%, Pain/stiffness 11%, Other 6% | 5.3 | rTKA using stepped, porous-coated, titanium metaphyseal sleeves (DePuy Synthes) | Re-revision, Implant survivorship, Re-operation, KSS, ROM, Radiographic assessment, Periprosthetic fracture rate | Nineteen knees (16.4%) required reoperation, most commonly for recurrent infection. Only one sleeve demonstrated radiographic evidence of failed osteointegration, but did not require revision. Two sleeves (1.5%) required removal and/or resection for recurrent infection. | This large retrospective series illustrates the utility of porous metaphyseal sleeves in revision TKA with a low rate of intraoperative complications, excellent osteointegration, and long-term fixation | Zone 2 fixation devices |
| Wilke | 2014 | Long-term survival of semi-constrained total knee arthroplasty for revision surgery | Journal of Arthroplasty | United States | Case series | IV | 234 | To examine survival rates and long-term clinical function of the semi-constrained modular fixed-bearing TC-III TKA implant used in the revision setting | Infection excluded, otherwise not clearly defined | 9 | rTKA using the semi-constrained modular fixed-bearing TC-III TKA implant | Re-revision, Implant survivorship, KSS, ROM, Pain | Forty repeat revisions were performed. 5-year survival was 91% and 10-year survival was 81%. Ninety percent of patients reported an improvement in their knee. | The semi-constrained implant used in revision knee arthroplasty has acceptable implant survival and functional outcomes in the long-term followup period. | Constraint |
| Wood | 2009 | Results of press-fit stems in revision knee arthroplasties | Clinical Orthopaedics and Related Research | Canada | Case series | IV | 135 | To report outcomes of press-fit uncemented stems and metaphyseal cement fixation in a selected series of patients undergoing revision total knee arthroplasty. | Instability 34%, Infection 25%, Aseptic loosening 21%, Osteolysis 19%, Fracture 1% | 5 | rTKA with press-fit uncemented stems and metaphyseal cement fixation (Genesis II; Smith&Nephew) | Re-revision, Implant survivorship, Radiographic assessment | Six patients (six knees) had revisions at a mean of 3.5 years (range, 1–8 years). Kaplan–Meier survivorship analysis revealed a probability of survival free of revision for aseptic loosening of 98% at 12 years. | Survivorship of press-fit stems for revision knee arthroplasty is comparable to reported survivorship of cemented stem revision knee arthroplasty. Radiographic analysis has shown continued satisfactory appearances regardless of constraint, stem size, and augmentations. | Cemented vs hybrid fixation |
| Xiong | 2021 | Outcome of Revision Surgery for the Idiopathic Stiff Total Knee Arthroplasty | Journal of Arthroplasty | United States | Retrospective comparative study | III | 202 | To report on postoperative outcomes of revision surgery for idiopathic stiff TKA | Idiopathic stiffness 53%, Non-idiopathic stiffness 47% | 4.2 | rTKA in patients with idiopathic stifness vs non-idiopathic stiffness | Re-revision, ROM | The overall revision surgery outcomes of the idiopathic stiffness cohort were worse than those of the non-idiopathic stiffness cohort with regard to maximum flexion (91.7deg vs 100.1deg, p=0.02) and flexion range of motion (ROM) (87.6 vs 97.1, p=0.01). In the idiopathic stiffness cohort, isolated tibial insert exchange demonstrated greater maximum flexion (p=0.06) and flexion ROM (p=0.07), and lower re-revision rates (16.7% vs 31.0% with component revision, p=0.01). | Isolated tibial insert exchange may be a preferred surgical treatment option in TKA patients with idiopathic stiffness | Isolated polyethylene exchange |
| Yapp | 2025 | Revision total knee arthroplasty using a fully cemented single-radius condylar constrained prosthesis has excellent ten-year survival and improvements in outcome measures: a cohort study of 358 implants | Bone & Joint Journal | United Kingdom | Case series | IV | 358 | To describe the long-term outcomes following rTKA using a fully cemented condylar-constrained knee arthroplasty | Component loosening 37%, Instability 35%, Infection 15%, Component wear 8%, Other 6% | 9.8 | rTKA with a fully cemented single-radius condylar constrained prosthesis | Implant survival, Re-revision, OKS, EQ-5D-3L | The overall ten year survival was 91.7% (95% CI 88.6 to 94.9); however, this differed significantly (p = 0.029) according to the indication for revision: aseptic loosening, 96.0% (95% CI 92.6 to 99.5); infection, 86.6% (95% CI 77.8 to 96.4); and instability, 85.3% (95% CI 77.4 to 93.9). There were significant (p < 0.001)improvements in PROMs at one year (OKS mean change (MC), 12 (95% CI 11 to 14); and EQ-5 D-3 L MC, 0.214 (95% CI 0.11 to 0.31)), which were sustained at the final follow-u p (OKS MC, 9 (95% CI 7 to 11) | RTKA using a cemented CCK arthroplasty had excellent ten-y ear survival and clinically meaningful improvements in PROMs. However, revision for instability or infection was associated with significantly worse survival and PROMs | Constraint |
| Yoon | 2019 | Rotating hinge knee versus constrained condylar knee in revision total knee arthroplasty: A meta-analysis | PLOS One | South Korea | Systematic review | II | 1177 | To evaluate the survival rates of short-term and midterm follow-up of RHK and CCK prostheses. The secondary end point was to compare results associated with clinical outcomes of RHK and CCK prostheses | Not defined; heterogeneous | Mean range 1.5-9.0 | rTKA with rotating hinge vs condylar constrained implants | Implant survivorship, Multiple PROMs, Complications | 87.4% of RHK and 75.0% of CCK prostheses survive at short-term (<5 years), while 81.3% of RHK and 83.8% of CCK prostheses survive at mid-term (5–10 years). CCK groups reported significantly better pain score (95% CI: 0.50 to 2.73; P = 0.005) and function score (95% CI: 0.01 to 2.00; P = 0.05) than RHK groups, however, these differeneces were likely to be imperceptible to patients and almost certainly below the minimum clinically important level. | RHK prostheses continue to be an option in complex RTKA with reasonable midterm survivorship | Constraint |
| Zanirato | 2018 | Metaphyseal sleeves in total knee arthroplasty revision: complications, clinical and radiological results. A systematic review of the literature | Archives of Orthopaedic & Trauma Surgery | Italy | Systematic review | II | 1079 | To summarise indications, complications, clinical and radiological mid-term results of metaphyseal sleeves in management of bone defects in rTKA. | Heterogeneous | 4 | rTKA with metaphyseal sleeves | Re-revision, Implant survivorship, Re-operation, Aseptic loosening, Infection, Intraoperative fracture rate | Sleeves allowed a stable metaphyseal fixation and osseointegration with an implant and sleeves aseptic survival rate of 97.7 and 99.2%, respectively. The incidence of PJI was 2.7 ± 2.4%. The estimated rate of reoperations and re-revisions were 14.2 ± 9.2 and 7.1 ± 4.8%, respectively | Metaphyseal sleeves represent a viable option in management of types IIb and III AORI bone defects in TKAr. Further high-quality log-term studies would better clarify complications, clinical and radiological results of this promising technique | Zone 2 fixation devices |
